# Development of Natural Plant Extracts as Sustainable Inhibitors for Efficient Protection of Mild Steel: Experimental and First-Principles Multi-Level Computational Methods

**DOI:** 10.3390/ma15238688

**Published:** 2022-12-06

**Authors:** Aisha H. Al-Moubaraki, Abdelkarim Chaouiki, Jamilah M. Alahmari, Wesam A. Al-hammadi, Ehteram A. Noor, Azza A. Al-Ghamdi, Young Gun Ko

**Affiliations:** 1 Department of Chemistry, Faculty of Sciences—Alfaisaliah Campus, University of Jeddah, Jeddah 21589, Saudi Arabia; 2 Materials Electrochemistry Laboratory, School of Materials Science and Engineering, Yeungnam University, Gyeongsan 38541, Republic of Korea; 3 Department of Chemistry, Faculty of Science and Humanities, Shaqra University, Dawadmi 11911, Saudi Arabia

**Keywords:** natural plant extract, corrosion protection, green inhibitor, electrochemistry, interfacial behavior, multi-level computational methods

## Abstract

In the present work, we present the superior corrosion inhibition properties of three plant-based products, *Fraxinus excelsior* (FEAE), *Zingiber zerumbet* (ZZAE), and *Isatis tinctoria* (ITAE), that efficiently inhibit the corrosion of mild steel in phosphoric acid. The anti-corrosion and adsorption characteristics were assessed using a combination of experimental and computational approaches. Weight loss, potentiodynamic polarization, and electrochemical impedance spectroscopy methods were used to evaluate the inhibitive performance of the inhibitors on the metal surface. Then, both DFT/DFTB calculations and molecular dynamic simulations were further adopted to investigate the interaction between organic inhibitor molecules and the metal surface. The protective layers assembled using the active constituents, such as carbonyl and hydroxyl groups, of the three plant-based products offer high electrochemical stability at high temperatures and robust protection against aggressive acidic solutions. All electrochemical measurements showed that the inhibition performance of extracts increased by increasing their concentration and improved in the following order: FEAE > ZZAE > ITAE. Further, these extracts worked as mixed-type inhibitors to block the anodic and cathodic active sites on the mild steel surface. Multi-level computational approaches revealed that FEAE is the most adsorbed inhibitor owing to its ability to provide electron lone pairs for electrophilic reactions. The experimental and theoretical results showed good agreement. These results indicate the possibility of replacing conventional compounds with natural substituted organic products in the fabrication of hybrid materials with effective anti-corrosion performance.

## 1. Introduction

Phosphoric acid (H_3_PO_4_) is second only to sulfuric acid (H_2_SO_4_) in terms of global production and has applications in the manufacture of fertilizers, food additives for animal rations, detergent powders, toothpaste, fire extinguishers, fuel components, medicines, water treatment, and agriculture. Phosphoric acid is commonly used to remove rust from iron and steel during metal surface treatments. It reacts with rust and turns the reddish-brown color of ferric oxide into a black ferric phosphate, which can be easily removed [1]. H_3_PO_4_ is also used to remove radioactive rust from the containers and pipes of nuclear reactors, and it is a major component of most commercial rust transformers. The chemical transformation or treatment of metal and alloy surfaces is of considerable economic importance [2]. Despite its importance in modern industry, metal degradation due to H_3_PO_4_ has become a major problem. To address this issue, various surface treatments have been developed to protect metallic surfaces in aggressive solutions. Recently, the use of organic inhibitors to protect the surfaces of metals, particularly steel, from highly corrosive acids has gained significance. The choice of inhibitor depends on the type of acid, its concentration, temperature, and velocity of flow, the presence of dissolved inorganic and organic substances, and the type of steel exposed in the acidic solution. Thus, it is necessary to consider the affinity, solubility, and adsorption capability of the synthesized inhibitors on the metal surfaces [3]. The development of potential corrosion inhibitors is essential to broaden the application of H_3_PO_4_ by reducing the corrosion rates of the equipment associated with it [4]. A variety of chemical species have been employed as corrosion inhibitors in phosphoric acid [5,6,7,8], but their importance has decreased due to their toxicity to humans and the environment. Therefore, the modern trend is towards exploring cheaper, readily available, and eco-friendly compounds of natural origin [9,10].

Natural extracts are the more environmentally friendly alternatives to conventional corrosion inhibitors. Extracts of plant parts, such as flowers, fruits, seeds, roots, peels, and bark, are considered rich reservoirs of natural chemical compounds that can be readily used to protect many metals and their alloys against various corrosive environments. Corrosion inhibitors from plant extracts have gained research interest because of their efficiency, availability, and easy and low-cost extraction [11,12,13]. Zanthoxylum Alatum and Psidium Guajava (guava) leaf extracts, rosemary and artemisia oils, apricot juice, black tea, and guar gum have been tested as green corrosion inhibitors for steel in H_3_PO_4_ solutions [14,15,16,17,18,19,20]. However, for a judicious selection of natural products for use as effective corrosion inhibitors, it is critical to understand the adsorption mechanisms involved. These reasons motivated us to explore the interfacial behavior of green corrosion inhibitors on steel surfaces.

In this study, three plant extracts derived from *Fraxinus excelsior* L. (FEAE), *Zingiber zerumbet* (ZZAE), and *Isatis tinctoria* L. (ITAE) were used to fabricate sustainable corrosion inhibitors, and their inhibition performance and interfacial behavior were evaluated. A brief description of each of these natural products is provided in the Supplementary Information (Figure S1) [21,22,23,24,25,26,27]. Due to their unique characteristics, such as strong affinity, good solubility and high adsorption capability, the synthesized extracts are considered as the promising eco-friendly corrosion inhibitors that have a significant effect on the physical and chemical processes of electrochemical corrosion behaviors by promoting the formation of a protective layer on the metal surface. Of course, FEAE, ZZAE, and ITAE represent a class of heterocyclic compounds containing one or more heteroatoms, polar functional groups, and aromatic rings. In addition to the π-conjugated molecular structure of the extracted inhibitors, their ability to serve as H-bond donors and acceptors might be key to the formation of organic layers on mild steel (MS) surfaces during adsorption.

The performance of these three plant extracts was investigated using electrochemical impedance spectroscopy (EIS), potentiodynamic polarization (PDP), and weight loss (WL) studies. In addition, the morphologies of the surfaces were evaluated using scanning electron microscopy (SEM) and Fourier-transform infrared (FT-IR) analysis. As mentioned above, the interfacial mechanism must be understood to elucidate the adsorption configuration of the inhibitor molecules and their interactions with the steel surface. Multilevel computational approaches based on density functional theory (DFT) and molecular dynamics (MD) simulations were performed to confirm the experimental results and verify the reactivity of the inhibitor molecules in terms of the charge-transfer ability and adsorption energy of the selected constituents.

## 2. Experimental

### 2.1. Preparation of Plant Extracts

Figure S2 shows the steps involved in the preparation of the corrosion inhibitors from the plant extracts. Dried *F. excelsior* seeds, *Z. zerumbet* roots, and *I. tinctoria* leaves were milled, and 50 g of each powder was boiled in 500 mL of deionized water for 2 h. The resulting solutions (extracts) were cooled and filtered to eliminate impurities. Following the filtration process, the extracts were dried in an oven at 60 °C to obtain crystalline residues (15.75 g, 8.45 g, and 8.65 g, respectively). Each precipitate was dissolved in 500 mL of deionized water, and the concentration of the extracts was expressed as *w*/*v* and was in the range of 0.005–3.00 g L^–1^. Table 1 illustrates the chemical structures of the major organic compounds present in the extracts.

### 2.2. Metal Specimens and Test Solutions

The MS specimens used for the corrosion tests had the following chemical composition (in %): 0.28 C, 0.22 Si, 0.73 Mn, 0.015 P, 0.006 Si, 0.007 N, and balance Fe. Before being placed in the test solutions for the experiments, MS rods of 5-cm in length and 1-cm in diameter were abraded using a 60–1200 grit size abrasive paper, cleaned thoroughly with deionized water and ethanol, and dried at laboratory temperature using an air stream. The aggressive solution used in this study, 2 M H_3_PO_4_, was prepared by diluting analytical grade H_3_PO_4_ (98% BDH) with deionized water. WL experiments were performed using a 50-mL solution, whereas electrochemical measurements with and without different concentrations of the inhibitors were conducted using a 100-mL solution.

### 2.3. WL Measurements

The pre-cleaned and weighed samples were placed in glass vessels containing the aggressive solutions with and without various concentrations of the plant extracts, which were placed in a thermostatic water bath for an 80-min immersion period. The MS specimens were removed from the test solutions at the end of each experiment, rinsed several times with deionized water and ethanol, dried in an air stream, and weighed on a digital scale with a 0.1 mg precision. For each assay, the weight loss (ΔW) was computed by subtracting the final weight from the initial weight, and the following equation was used to calculate the MS corrosion rate (*CR_WL_*, in g cm^−2^ min^−1^).

(1) $$CR_{WL}=\frac{\Delta W}{A\times t}$$

where *t* is the exposure duration (min) and *A* is the exposure area of the specimen. Using the *CR* values, the inhibition efficiency (*IE_WL_*%) was estimated using the following relationship:

(2) $$IE_{WL}\%=\left(1-\frac{CR_{WL}}{CR_{WL}^{o}}\right)\times 100$$

where $CR_{WL}$ and $CR_{WL}^{o}$ represent the *CR* with and without various concentrations of the plant extracts, respectively.

Each recorded value was the result of duplicate experiments, and the aggressive acid solution was open to the air.

### 2.4. Evaluation of Electrochemical Corrosion

The electrochemical experiments were carried out using a 250-mL one-compartment cell containing three electrodes connected to an ACM Gill AC instrument model 1649 with a computer. The three-electrode cell included an MS sample of 0.746 cm^2^ embedded in Teflon using an epoxy resin as the working electrode and high-purity platinum foil as the counter electrode, coupled with a silver chloride electrode (Ag/AgCl/KCl_sat_) as the reference electrode. EIS measurements were conducted at an open circuit potential in a frequency range of 0.1 Hz–30 kHz with a signal amplitude perturbation of 10 mV. The impedance responses were fitted to the appropriate equivalent circuit using the simulation program ZSimDemo (3.20 software), and corrosion parameters, such as solution resistance (*R_s_*), polarization resistance (*R_p_*), and double-layer capacitance (*C_dl_*), for various electrochemical processes occurring at the metal surface were estimated. Later, PDP scans were performed in the potential range from –700 to –200 mV with respect to the open-circuit potential at a scan rate of 1 mV s^–1^. The corrosion current density ($i_{corr}$), corrosion potential $\left(E_{corr}\right)$, and Tafel constants ($\beta_{a\;}\mathrm{and}\;\beta_{c}$) were obtained by analyzing the polarization curves using the ACM Gill software (model 1649). All the electrochemical tests were performed in stagnant solutions in the open air.

The adsorption performance of the plant extracts was evaluated using the polarization resistance and corrosion current density values with ($R_{p}$ and $i_{corr}$) and without ($R_{p}^{o}$ and $i_{corr}^{o}$) the plant extracts according to Equations (3) and (4), respectively.

(3) $$IE_{R}\%=\left(1-\frac{R_{p}^{o}}{R_{p}}\right)\times 100$$

(4) $$IE_{i}\%=\left(1-\frac{i_{corr}}{i_{corr}^{o}}\right)\times 100$$

### 2.5. Surface Characterization

The surface morphologies of MS immersed in H_3_PO_4_ solutions free of and containing low (0.025 g L^–1^) and high (2.5 g L^–1^) concentrations of each plant extract were examined using scanning electron microscopy (SEM) at the Center of Nanotechnology, King Abdulaziz University. The corrosion products were subjected to FT-IR analysis to confirm the adsorption of the inhibitor molecules on the MS surface, and the FT-IR spectra of the inhibitor powder and corrosion products were compared. The KBr disk technique was employed to record the FT-IR spectra using a Perkin-Elmer spectrophotometer (Frontier, Waltham, MA, USA), with a 400–4000 cm^–1^ range. The plant extract powder, which was mixed with KBr and compressed into a disk, was used as the initial sample for the FT-IR characterization. The second sample consisted of the corrosion products of an MS surface that had been immersed for three days in a 2 M H_3_PO_4_ solution containing 2.5 g L^–1^ of each plant extract, rinsed with deionized water, dried in air, scraped with a small quantity of KBr, and compressed into disks.

Unless otherwise specified, all experimental measurements were carried out in stagnant solutions at 30 °C using a thermostatic water bath (LOBT-B12) with temperature ranging from 0 °C to 99 °C and temperature stability of ±0.5 °C.

### 2.6. Theoretical Details and Models

The quantum chemical (QC) properties and interfacial behavior of the major compounds present in the plant extracts on metal surfaces were investigated using multi-level computational model. Density functional theory (DFT), density functional-based tight-binding (DFTB), and molecular dynamics (MD) simulations were used to highlight and correlate the molecular features of the extract molecules with their adsorption mechanisms. First, the charge transfer behavior of plant extracts were evaluated using DFT method at B3LYP of Gaussian 16 as described elsewhere [28]. All experiments were performed using the 6-311G++ (d,p) basis set in the aqueous phase, as defined by the solvent model density (SMD). The corresponding QC properties, such as the HOMO and LUMO energies, energy gap (ΔE), electronegativity (χ), and fraction of transferred electrons (ΔN), were computed as detailed elsewhere [29,30]. Furthermore, based on conceptual DFT, other functions such as the density of states (DOS), electron localization function (ELF), and electrostatic potential (ESP) were analyzed to characterize the electron distribution, predict the reactive sites, and analyze the reactivity. DFTB computations were then performed to explore the interfacial behavior of the inhibitor molecules on the metallic surface [31] and model the adsorption mechanisms in large and complex systems using the Material Studio software package (version 6.0) and the trans3d Slater–Koster library [32], combined with an empirical dispersion correction within the self-consistent charge (SCC) formalism [33], respectively. All systems were optimized using an SCC tolerance of 0.05 kcal mol^–1^ for energy, 0.5 kcal mol^–1^ Å^–1^ for force, and 0.001 Å for maximum displacement. Broyden mixing and small thermal smearing were used to speed up the SCC convergence [34]. The Fe surface was fully constructed along the (110) plane and modeled as a periodic array of four layers with a 10 × 9 supercell and a vacuum slab of 30 Å in the z-direction. K-points of 3 × 3 × 1 were selected to simulate the adsorption process. For each system, we considered a parallel adsorption model in which the two bottom layers of the slab were constrained, while the inhibitor and the upper half of the slab were fully relaxed. Subsequently, the adsorption energy (*E_ads_*) between the iron surface and any given inhibitor can be defined as follows [35,36]:

(5) $$E_{ads}=E_{surf+inh}-\left(E_{surf}+E_{inh}\right)$$

where *E_surf+inh_* is the total energy of the inhibitor/Fe(110), *E_surf_* is the energy of the clean iron slab, and E_inh_ is the energy of the isolated inhibitor.

To simulate the effect of temperature on the synergistic adsorption mechanisms of the inhibitor molecules in aqueous solutions, the Forcite module in Materials Studio was used to perform the MD calculations [37]. As an ideal surface, Fe(110) was cleaved with a periodic structure. A simulation box of 39.93 Å × 39.93 Å × 77.04 Å comprising a 10 × 10 iron slab, an aqueous phase (H_2_O, H_3_O^+^, PO_4_^3−^, and the inhibitor molecule), and a vacuum layer was created. MD calculations were preceded by geometrical optimization; then, four inhibitor molecules and the corresponding molecular ions were allowed to interact with the iron surface. Subsequently, all MD simulations were performed using a COMPASS II force field with an NVT ensemble at 298 K with a fine calculation accuracy and a simulation time of 500 ps with a step time of 1 fs [38,39,40]. The interaction of the organic compounds with the iron surface in a corrosive solution was estimated using the following formula:

(6) $$E_{inter}=E_{total}\;–(E_{surf+sol}+E_{inh+sol})+E_{sol}$$

where *E_total_*, *E_surf+sol_*, *E_inh+sol_*, and *E_sol_* are the energies of the simulation system, iron slab and solution, inhibitor molecule and solution, and solution.

## 3. Results and Discussion

### 3.1. Assessment of Inhibitors Concentration Effect

#### 3.1.1. Weight Loss Measurements

The short-term corrosion experiment results (Table 2) show that the corrosion rate decreases with increasing plant extract concentration, suggesting that the inhibition performance (IE_WL_%) was significantly enhanced by the addition of plant extracts to the electrolyte (Figure S3a,b). The inhibition performance had maximum values of 95.2%, 92.8%, and 91.8% at 3.0 g L^–1^ for FEAE, ZZAE, and ITAE, respectively, indicating that they efficiently inhibit corrosion in 2 M H_3_PO_4_ solutions at 30 °C. The inhibition is achieved by the accumulation and adsorption of the plant extract molecules on the MS surface. In general, the plant extract molecules suppress the dissolution of the metal by forming a protective layer adsorbed on its surface and preventing the corrosion-causing ions from reaching it.

Figure S3c presents the inhibition coefficient (*γ*) as a function of the inhibitor concentration. γ was calculated using Equation (7) [41].

(7) $$\gamma =\frac{CR_{WL}^{o}}{CR_{WL}}$$

As shown in the figure, γ depends on both the plant extract type and concentration. In the case of FEAE, *γ* increases gradually with concentration and reaches a maximum value of 20.8 at 3.0 g L^–1^. However, for ZZAE and ITAE, the γ values increase very slightly until 0.25 g L^–1^, after which they increase sharply and reach the maximum values of 13.9 and 12.8, respectively, at 3.0 g L^–1^. Based on these results, the inhibition efficiencies of the plant extracts can be arranged as follows: FEAE > ZZAE > ITAE.

#### 3.1.2. EIS Coupled with Equivalent Circuit Model

**Figure 1:**
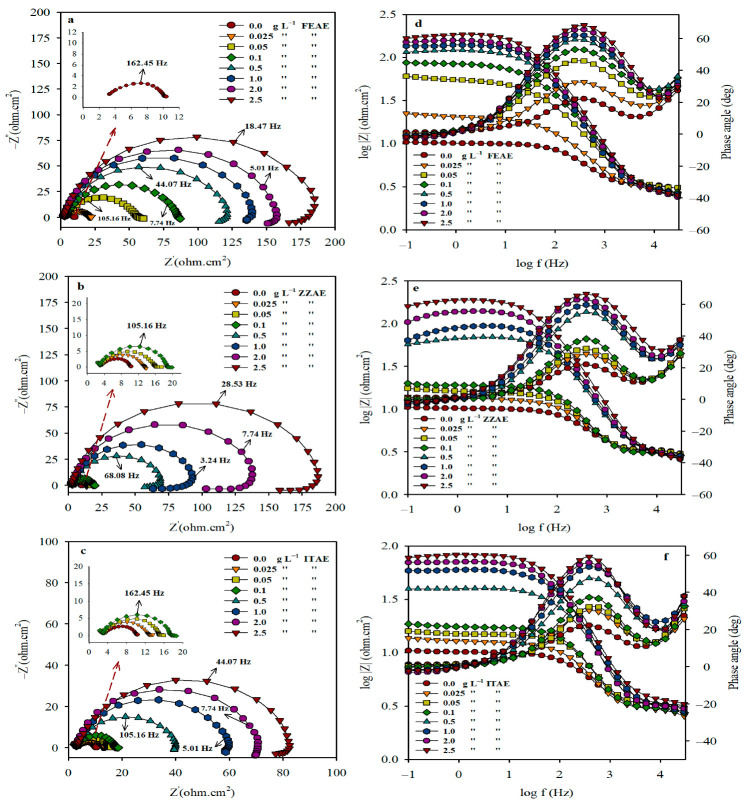
Nyquist (**a**–**c**), Bode impedance modulus and phase angle plots (**d**–**f**) for mild steel corrosion in 2 M H_3_PO_4_ with and without different concentrations of the studied plant extracts at 30 °C.

The data on the resistive and capacitive behaviors of the plant-based extracts at the interface were obtained using EIS [42,43]. Figure 1a–c shows the impedance responses of FEAE, ZZAE, and ITAE for MS corrosion in 2 M H_3_PO_4_ solutions. The semicircular Nyquist plots reveal that the charge-transfer process occurs during the dissolution of the metal. The semicircle appears to deviate from the ideal capacitive behavior, corresponding to the frequency dispersion of the interfacial impedance resulting from the surface heterogeneities and roughness factors [44]. However, the semicircle gradually improves with increasing inhibitor concentration. The size of the loop in 2 M H_3_PO_4_ increases with the inhibitor concentration, indicating that the inhibitor molecules in the solution hindered the surface transfer of the aggressive species. Therefore, the corrosion inhibition can be attributed to the adsorption of inhibitor molecules on the metal surface, which can block the corrosion sites. At high concentrations of the investigated plant extracts, an induction loop emerges at low frequencies, which could be attributed to the relaxation of the adsorbed inhibitor molecules or the redissolution of the passivated surface [45].

Figure 1d–f depicts the Bode impedance moduli of log(f)–log(Z) obtained for MS with and without various concentrations of the inhibitors. The Bode moduli show linearity at intermediate frequencies, which is more noticeable in the presence of the inhibitors, showing higher slopes than the free solution [46]. The linearity increases with the inhibitor concentration, indicating a higher protection ability at higher inhibitor concentrations. The increase in the absolute impedance at low frequencies further proves that increasing the inhibitor concentration leads to increased protection, which is linked to the adsorption of the inhibitor molecules on the metal surface.

Figure 1d–f also depicts the Bode phase angle plots obtained for MS with and without various concentrations of the inhibitors. The log(f)-phase angle plots illustrate only one peak, confirming the presence of a single time constant [47]. The increase in the peak height with inhibitor concentration can be associated with a predominantly capacitive response at the metal–solution interface due to increased adsorption of the inhibitor molecules on the metal surface and consequent decline in metal dissolution [48].

Pure electric models can explain impedance behavior and can be used to decouple electrochemical processes at the metal-solution interface in order to determine the physical and chemical parameters of the electrochemical system under study. By fitting the proposed electrical circuit to the experimental data, as shown in Figure S4, the appropriate parameters for the electrical circuit can be obtained. The circuit in Figure S4a was used to characterize the systems containing 0.025–0.1 g L^–1^ or no inhibitors. A more complex electrical circuit, as shown in Figure S4b, was used for the system containing 0.5–2.5 g L^–1^ of the inhibitors. The two equivalent circuits consist of the following main elements: *R_s_* represents the solution resistance, *CPE_dl_* (*Q_dl_*, *n*) is a constant phase element, instead of pure capacitors, that represents numerous non-homogeneities characteristic of corroding electrodes, and *R_p_* is the polarization resistance with barrier-type characteristics, including the charge transfer (*R_ct_*), diffuse layer(*R_d_*), accumulation (*R_a_*), inductance (*R_L_*), and film (*R_f_*) resistances [49,50].

Using the equivalent circuit model shown in Figure S4b, the polarization resistance *R_p_* can be calculated using the following formula [51]:

(8) $$R_{p}=\frac{R_{ct}\times R_{L}}{R_{ct}+R_{L}}$$

The double-layer capacitance (*C_dl_*) is defined as follows:

(9) $$C_{dl\;}=\sqrt[{n}]{Q_{dl}\times R_{p}^{1-n}}$$

where *Q_dl_* and *n* are the major components of CPE. *n* is a *CPE* exponent which provides information about the surface inhomogeneity. For a real electrode processes, its value is often between 0 and 1. *Q_dl_* is the non-ideal double-layer capacitance separating the metal–electrolyte interface. Table 3 displays the estimated electrochemical impedance parameters and calculated inhibition performance (*IE_R_*%) values with and without various concentrations of the inhibitors. Chi-square (χ2) values of the order 10^−3^ supported the validity of the proposed circuits, which was also demonstrated by the consistency of the experimental data (Figure 1a–c) and the fitted curves (Figure S4). The recorded *R_s_* values demonstrated that the solution resistance did not change considerably with increasing inhibitor concentration, indicating that the plant extracts have good conductivity in 2 M H_3_PO_4_. Moreover, it is commonly known that the impedance of a *CPE* is defined as [43]:

(10) $$Z_{CPE}=\frac{{\left(j\omega \right)}^{-n}}{Q}$$

where *j* and *ω* denote the imaginary number and angular frequency, respectively, and *n* and *Q* are *CPE* parameters. For the corrosion of metals in aggressive solutions, *n* is used to explain the electrochemical behavior of *CPE*. *Z_CPE_* represents a resistive behavior with *R* = 1/*Q* for *n* = 0 and a capacitive behavior with *C* = *Q* for *n* = 1. The observed value of *n* in this work ranges between 0.8 and 1.0, indicating that *CPE* has a pseudo-capacitive response in both the inhibited and non-inhibited conditions [52].

Table 3 indicates that the values of *R_p_* increase, whereas those of *C_dl_* decrease significantly, with increasing inhibitor concentration. Larger impedances result in lower interface activities, i.e., fewer electrons or ions passing through the interface, thereby lowering the corrosion rates. The decrease in *C_dl_* can be attributed to a decrease in the value of the double-layer dielectric constant or electrode surface area, or an increase in the double-layer thickness [52]. As the surface area of the electrode was constant during all the measurements, the decrease in *C_dl_* can be attributed to the replacement of the pre-adsorbed water molecules on the metal surface with the inhibitor species, which have a lower dielectric constant, and/or an increase in the thickness of the electrical double layer, indicating that the inhibitor molecules act via adsorption at the metal/solution interface and reduce the MS corrosion rates. The *R_p_* values increase with increasing inhibitor concentration, leading to an enhanced *IE_R_%*. According to the *IE_R_%*, the plant extracts can be arranged as follows: FEAE > ZZAE > ITAE. This arrangement is consistent with the results obtained from the WL measurements.

#### 3.1.3. Polarization Behavior

**Figure 2:**
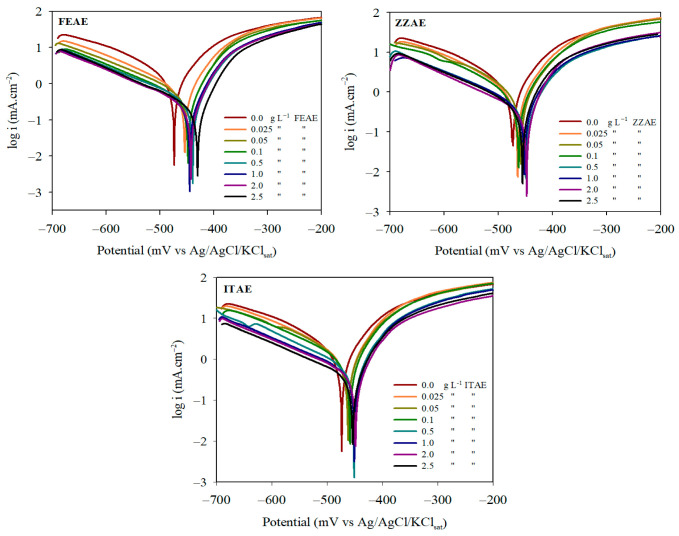
PDP curves for mild steel corrosion in 2 M H_3_PO_4_ with and without different concentrations of the studied plant extracts at 30 °C.

Potentiodynamic scans were performed to determine the effects of the plant extracts on the anodic dissolution of the metal and cathodic hydrogen evolution. The PDP curves of MS in 2 M H_3_PO_4_ with and without the three plant extracts (Figure 2) reveal that the addition of the three plant extracts affects the anodic metallic dissolution and cathodic hydrogen evolution processes without the common characteristics of the modified curves. Essentially, the presence of the plant extracts displaces both the anodic and cathodic polarization curves towards lower current densities, that is, towards lower rates of MS corrosion This effect is more pronounced with increasing plant extract concentration. The electrochemical potentio-kinetic parameters of MS corrosion, determined using the Tafel extrapolation method, are presented in Table 4. These electrochemical parameters consist of the corrosion potential (*E_corr_*), corrosion current density (*i_corr_*), slopes of the anodic and cathodic branches (*β_a_* and *β_c_*), and inhibition efficiency (*IE_i_*%). A general comparison is presented in the form of the polarization resistance (*R_p_*) by employing the Stern–Geary equation [53]:

(11) $$R_{p}=\frac{B^{\prime }}{i_{corr}}$$

where $B^{\prime }=(\beta_{a}\left|\beta_{c}\right|$)/(2.303($\beta_{a}+\left|\beta_{c}\right|)$.

Obviously, the values of *E_corr_* with the presence of the plant extracts were shifted to more positive values in comparison to that in the absence of inhibitors, which indicates that the inhibition for anodic iron dissolution is stronger than the cathodic hydrogen evolution reaction. This indicates that the formed protective layers can cause a significant reduction in the amount of corrosive species reaching the metallic substrate. Thus, the steel surface treated by the plant extracts exhibited higher corrosion resistance than the untreated samples. In addition, based on the general criterion in the corrosion field [7,19], a positive shift of *E_corr_* less than 85 mV after adding inhibitors with respect to the corrosion potential of the blank reflects the mixed-type behavior of inhibitors. As seen in Figure 2 and Table 4, the magnitude of the *E_corr_* change is significantly less than 85 mV and the maximum displacement obtained was 42, 27, and 25 mV for FEAE, ZZAE, and ITAE, respectively, with respect to 2 M H_3_PO_4_ without inhibitors, suggesting a mixed-control behavior of the plant extracts. *β_a_* and *β_c_* changed slightly with increasing extract concentration, confirming that the plant extracts functioned via a mixed adsorption mechanism. Moreover, the presence of plant extracts in the corrosive medium does not change the anodic and cathodic mechanisms, indicating that the inhibitors act by simply blocking the active sites on the metallic surface [54]. Increasing the inhibitor concentration reduces the *i_corr_* and subsequently increases the *IE_i_*%. Table 4 indicates that the addition of the inhibitors to the electrolyte until a value of approximately 2.5 g L^–1^ increases the *IE_i_*%, which may have significant implications. At high concentrations, more inhibitor species are present, and a greater surface coverage is achieved. Polarization resistance, *R_p_*, recorded in Table 4, is an important kinetic parameter. Increasing values of *R_p_* with an increasing extract concentration imply lower corrosion metal dissolution rates. From the inhibition efficiency (*IE_i_*%) data, the studied inhibitors can be arranged as follows: FEAE > ZZAE > ITAE.

This arrangement is consistent with the results obtained from the EIS and WL measurements.

### 3.2. Morphological Analysis

**Figure 3:**
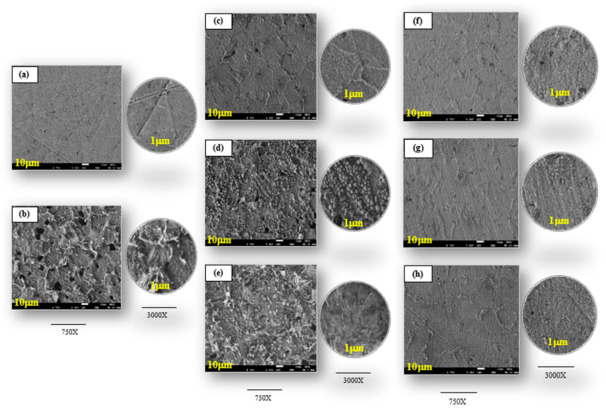
SEM micrographs of mild steel (**a**) only surface abrasion, (**b**) after immersion in 2 M H_3_PO_4_ without inhibitors, with 0.025 g L^−1^ of (**c**) FEAE, (**d**) ZZAE, (**e**) ITAE, and with 2.5 g L^−1^ of (**f**) FEAE, (**g**) ZZAE, and (**h**) ITAE.

SEM images of the polished and corroded MS surface without and with low (0.025 g L^–1^) and high (2.5 g L^–1^) concentrations of the plant extracts are shown in Figure 3a–h. The freshly polished MS surface was smooth with minor scratches due to the abrasive SiC paper (Figure 3a). However, the uninhibited MS surface (Figure 3b) was severely damaged and became rough, with a large number of irregular pits distributed over the metal surface, owing to the aggressive attack of the corrosive solution. In contrast, the appearance of the MS surface was different after the addition of the plant extracts, and the inhibition efficiency varied with the inhibitor concentration. With the addition of 0.025 g L^–1^ of each plant extract, the corrosion was significantly diminished, along with the number and depth of the pits (Figure 3c–e). The adsorption of the inhibitor species on the MS surface and the creation of a protective layer on the electrolyte/electrode interface can explain these phenomena. By increasing the concentration (2.5 g L^–1^) of the plant extracts (Figure 3f–h), the MS surface became more uniform, smoother, and brighter, with visible polishing scratches, as the inhibitors’ dense protective layer prevented the corrosion agents from acting. These results confirm the findings of the WL, EIS, and PDP measurements and further explain the high inhibition efficiency of the studied plant extracts at high concentration (2.5 g L^–1^).

### 3.3. FT-IR Analysis

**Figure 4:**
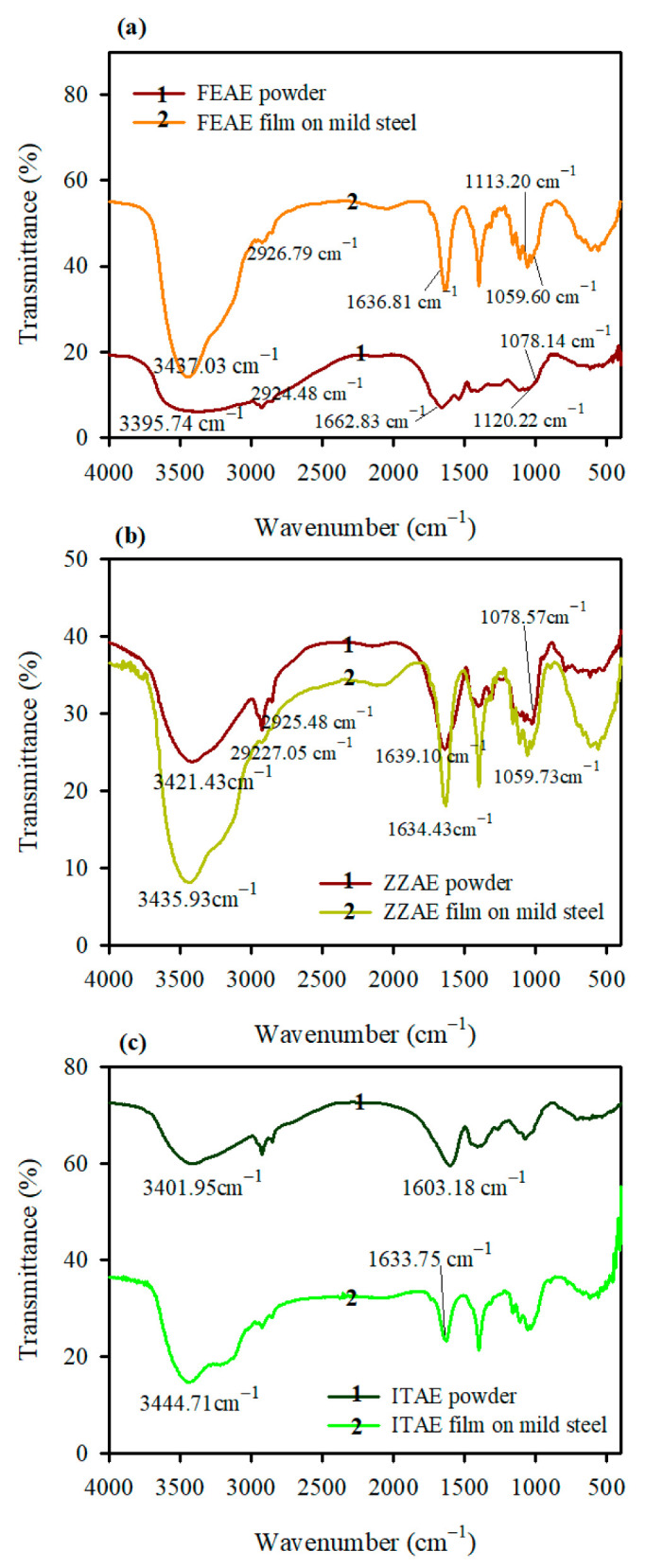
FT-IR spectra of (**a**) FEAE, (**b**) ZZAE, and (**c**) ITAE powder and film formed on the mild steel surface after immersion in 2 M H_3_PO_4_ containing 2.5 g L^−1^ of the studied plant extracts.

The application of FT-IR spectroscopy to identify and determine the type of organic functional groups contained in the plant extract that can adsorb by establishing bonds with the steel surface is well recognized [43]. Figure 4 shows the FT-IR spectra of the plant extracts and the film formed on the MS surface after its immersion in the electrolyte containing 2.5 g L^–1^ of the plant extracts. The prominent peaks are listed in Table 5. Both changes in vibrational frequencies and band intensities are observed, supporting the adsorption of the studied plant extracts on the MS surface from H_3_PO_4_ solution. In the case of FEAE, a broad band was recorded at 3395.74 cm^−1^ due to hydrogen bond formation between OH and carbonyl groups in FEAE species. While in the film formed on the metal surface, a sharp intense absorption band at 3437.03 cm^−1^ was observed, confirming the formation of iron hydroxide between the metal and the adsorbed layer. It also confirms the disappearance of hydrogen bonding between OH and carbonyl groups of the FEAE species. In addition, the weakness of ν (C=O), ν (C=C), and ν (C-H) vibrations with their shifts compared with plant extract powder supports the interaction of the plant extracts with the MS surface. The weakness of band intensities of the film formed on the MS surface revealed a strong interaction between MS and plant extracts species, mainly through electrostatic interaction, as well as the formation of a chemical bond between Fe and plant extracts species.

### 3.4. Adsorption Isotherms

**Figure 5:**
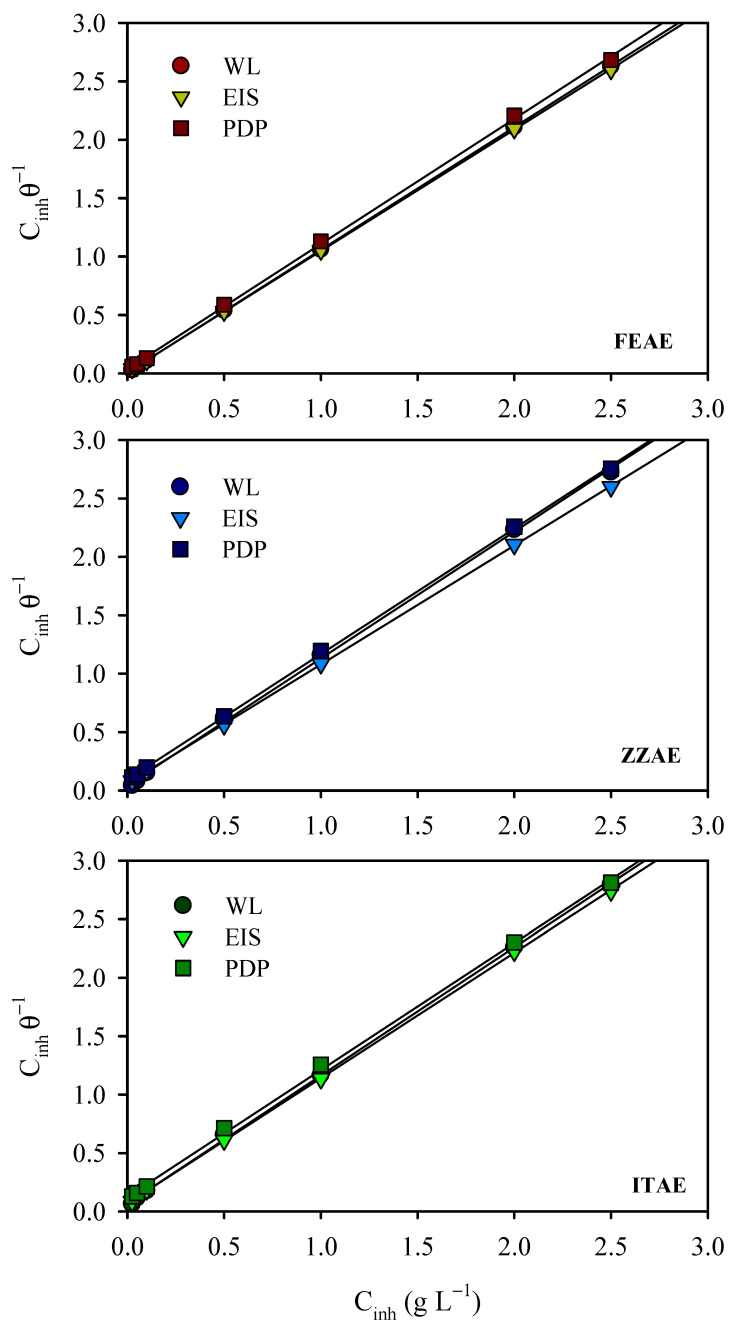
Langmuir adsorption isotherm for the studied plant extracts on mild steel from 2 M H_3_PO_4_.

During the adsorption process, a higher degree of surface coverage by the inhibitor is critical for effective corrosion protection. Furthermore, the interfacial mechanism of the corrosion inhibitors depends on the direct or indirect adsorption of the inhibitor particles onto the metal surface, which reduces the direct contact between the aggressive species and the metal surface. The interfacial behavior was evaluated using the Langmuir [19,55], Temkin, Freundlich [56,57], Frumkin [58,59], and Flory-Huggins [60,61] adsorption isotherms, whereby the amount of adsorbate (molecules/ions of inhibitors) on the absorbent (metallic surface) was estimated at a constant temperature. The mathematical formulae of these isotherms and the related descriptions are listed in Table S1. The surface coverage ($\theta =\frac{IE\%}{100}$) values obtained from the results of the WL, EIS, and PDP measurements were best fitted to the Langmuir adsorption model (Equation (12), Figure 5). Estimating correlation coefficients by fitting the different results of some isotherm models is depicted in Table S2. Figure 5 shows straight lines with high correlation coefficients (*r*^2^ ≈ 1) and slopes ranging from 1.02 to 1.10, confirming that the inhibitor molecules were adsorbed on the steel surface in a single layer according to the Langmuir adsorption isotherm. The deviation of the slopes from unity (Table 6) indicates interactions between the adsorbate molecules. These adsorbed species may interact mutually, either by attraction or repulsion. Certain components of the plant extract may be adsorbed on the anodic or cathodic regions, leading to the deviation of the slopes from unity [47]. The intercept of the lines represents the adsorption equilibrium constant (*K_ads_*) and is related to the Gibbs free energy change of adsorption (Δ*G_ads_*) by the following equation [62]:

(12) $$K_{ads}=\frac{1}{C_{H_{2}O}}\exp\left(-\frac{\Delta G_{ads}}{RT}\right)$$

where $C_{H_{2}O}$ is the water molecule concentration in the double layer in g L^–1^, *R* is the universal gas constant (8.314 J mol^–1^ K^–1^), and *T* is the absolute temperature in *K*. Table 6 shows the estimated Langmuir parameters and Gibbs free energy changes for adsorption. The *K_ads_* values revealed that FEAE has the highest adsorption ability for the steel surface. The greater the *K_ads_*, the stronger and more efficient the adsorbed layer on the steel surface and the better the surface coverage [63]. The negative values of Δ*G_ads_* imply favorable adsorption of the extract species on the steel surface and the practicability and spontaneous nature of the adsorption process, especially the FEAE. The calculated values of Δ*G_ads_* lie between –22.65 and –29.77 kJ mol^–1^, confirming that the extract species are adsorbed on the MS surface through both physisorption and chemisorption (mixed mode) mechanisms [50].

### 3.5. Effect of Temperature on Electrochemical Stability and Thermodynamic Behavior

**Figure 6:**
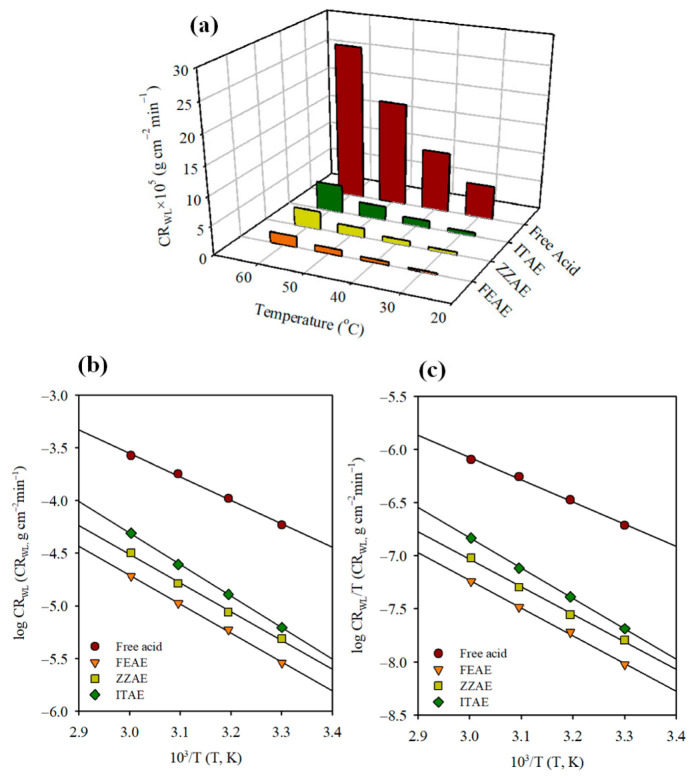
(**a**) Variation of CR_WL_ with temperatures, (**b**) Arrhenius and (**c**) transition state plots for mild steel corrosion rates (CR_WL_) in 2 M H_3_PO_4_ with and without 2.5 g L^−1^ of the studied plant extracts.

The effect of temperature on the inhibited acid/metal systems is complex. It has a significant influence on the electrochemical corrosion reactions due to multiple changes on the metal surface, such as inhibitor molecule desorption, rapid etching, and breakdown and/or rearrangement of the inhibitor molecules [64,65]. Consequently, temperature changes influence metal dissolution, inhibitor adsorption/desorption, and inhibitor efficiency. Based on the short-term experimental results obtained from the WL studies, the influence of temperature on the corrosion rates and inhibition behavior of MS in 2 M H_3_PO_4_ with and without 2.5 g L^–1^ of plant extracts is shown in Figure 6a and Table S3. The results show that the corrosion rates increase for both the uninhibited and inhibited acid solutions, and the inhibition performance decreases slightly with increasing temperature. The increase in the MS corrosion rates can be attributed to the increase in the average kinetic energy of the interacting species. Nevertheless, the plant extracts remained effective corrosion inhibitors for mild steel up to 60 °C (92.8%, 88.1%, and 81.6% for FEAE, ZZAE, and ITAE, respectively). However, at a given temperature, the electrochemical stability of the investigated plant extracts could be ordered as follows: FEAE > ZZAE > ITAE. These results indicate that the plant extracts are efficient at the range of temperature studied.

To assess the strength of the plant extracts as inhibitors over the studied temperature range, $\Delta IE_{\left(30^{{}^{\circ}}-60^{{}^{\circ}}\right)C}\%$ were calculated as follows:

(13) $$\Delta IE_{\left(30^{{}^{\circ}}-60^{{}^{\circ}}\right)C}\%=IE_{\left(30^{{}^{\circ}}\right)}-IE_{\left(60^{{}^{\circ}}\right)}$$

where $\Delta IE_{\left(30^{{}^{\circ}}-60^{{}^{\circ}}\right)C}\%$ represents the overall decline in the *IE*% with increasing temperature from 30 °C to 60 °C and may provide a good criterion for the stability and the performance of the inhibitors with temperature. The decrease in the inhibition efficiency was between 2.3% (FEAE) and 7.8% (ITAE), demonstrating the excellent corrosion protection of the plant extracts, particularly FEAE. These results indicate the formation of an organic protective film on the MS surface in which strong donor–acceptor interactions become important.

In addition, it is worth noting that the thermodynamic characteristics of the inhibitors is crucial for a better understanding of the adsorption process at the steel/solution interface. The Arrhenius and transition state equations were used to evaluate the temperature dependence of the rate constant [66]. In corrosion science, the linear form of Arrhenius and transition state-equations can be given as follows:

(14) $$log\left(\mathrm{rate}\right)=logA-\frac{E_{a}}{\;2.303RT}$$

(15) $$log(\frac{rate}{T})=\left[\log(\frac{R}{N_{A}h})+\frac{\Delta S^{*}}{2.303R}\right]-\frac{\Delta H^{*}}{2.303RT}$$

where $E_{a}$ is the apparent activation energy, *A* is the pre-exponential factor, and *R* is the universal gas constant (8.314 J mol^−1^ K^−1^). $\Delta H^{*}$ and $\Delta S^{*}$ are the enthalpy and entropy changes of activation for the complex formation in the transition state, *h* is Plank’s constant, and $N_{A}$ is Avogadro’s number. Based on the WL studies, Figure 6b,c represents the regression of log (rate) and log (rate/T) with 1/T for MS without and with 2.5 g L^–1^ of the plant extracts. The rate of metal dissolution is determined based on corrosion rate obtained from WL method. Straight lines are observed in both cases, having slopes (−Ea2.303R) and (−ΔH*2.303R) and intercepts *logA* and $\left[log\frac{R}{N_{A}h}+\frac{\Delta S^{*}}{2.3033R}\right]$, as depicted in Figure 6b,c, respectively, from which the values of $E_{a}$, *A*,$\;\Delta H^{*}$, and $\Delta S^{*}$ were evaluated, as listed in Table 7.

The $E_{a}$ values (42.65 kJ mol^–1^) in H_3_PO_4_ without inhibitors are consistent with the values reported in literature for steel in the same acidic medium [67]. However, all inhibited systems showed greater $E_{a}$ values compared to uninhibited systems, indicating a strong inhibitory action for the studied plant extracts by increasing the energy barrier for the corrosion process [66,68].

The pre-exponential factor, *A*, in the Arrhenius-type relationship for the corrosion behavior and heterogeneous reactions is related to the number of active centers. Assuming energetic surface homogeneity, these active regions had different energies. The tendencies of dissimilarity in *A* and $E_{a}$ are comparable, implying that they are positively correlated [69].

The $\Delta H^{*}$ values exhibit a trend similar to that of the $E_{a}$ values (Table 7), indicating a higher protection efficiency. The positive values of $\Delta H^{*}$ for the uninhibited and inhibited solutions reflect the endothermic nature of the MS dissolution process. Generally, $\Delta H^{*}$ approaches 100 kJ mol^–1^ for the chemisorption process and less than 40 kJ mol^–1^ for the physisorption process. It can be seen that the $\Delta H^{*}$ values are larger than the expected physical adsorption but smaller than the one for chemical adsorption, indicating that the adsorption mechanism of three plant extracts at the studied temperatures involve both physisorption and chemisorption. In fact, chemisorption of the plant extracts occurs due to the formation of links between the d orbital of iron atoms, involving the displacement of water molecules from the metal surface, and the lone electron pairs present on the N and O atoms of the extract molecules. This was confirmed by DFT and DFTB calculations. It is noteworthy that the difference between $E_{a}$ and $\Delta H^{*}$ is approximately constant for all the systems and has an average value equal to *RT* as per Equation (16), suggesting that MS corrosion is a unimolecular reaction [66].

(16) $$E_{a}-\Delta H^{*}=RT$$

The uninhibited solution has negative $\Delta S^{*}$ values, which become less negative with the addition of the plant extracts (Table 7). Grigoryev et al. [70,71] have discussed this phenomenon. The transition state of the rate-determining recombination phase reflects a more orderly arrangement than the initial state in free acid solutions, resulting in the negative value of $\Delta S^{*}$. In the presence of inhibitors, the rate-determining step is the discharge of hydrogen ions to produce adsorbed hydrogen atoms. Because the surface is covered with inhibitors molecules, the discharge of hydrogen ions at the solution–metal interface is hampered, causing the system to shift from a more ordered to a random state, resulting in a less negative $\Delta S^{*}$.

### 3.6. Computational Approaches

#### 3.6.1. Molecular Electronic Properties of Isolated Molecules

**Figure 7:**
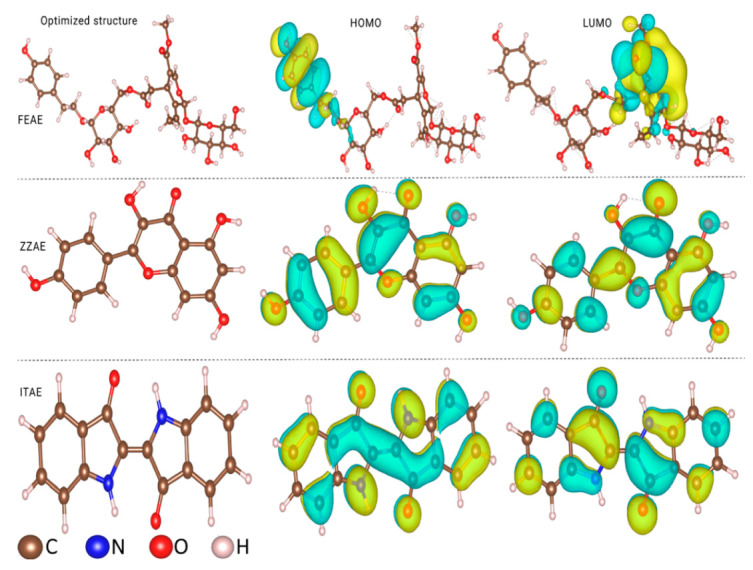
Optimized geometries and MO distributions of the major components in natural plant extracts.

To shed light on the electronic structures of the main compounds present in the plant extracts, we first modeled the isolated molecules, and their density distribution was described by frontier molecular orbital (FMO) theory. The optimized geometries and FMO distributions of FEAE, ZZAE, and ITAE are shown in Figure 7. The HOMO and LUMO were evenly distributed throughout the various moieties of the ZZAE and ITAE molecules, whereas the FEAE molecules had a less concentrated distribution of HOMO and LUMO, which were primarily concentrated around specific moieties, namely the 4-(2-hydroxyethyl)phenol and hydroxy-2-(methoxycarbonyl)cyclohexane. The presence of N and O atoms, as well as benzene rings, can affect the molecular orbital distributions.

**Figure 8:**
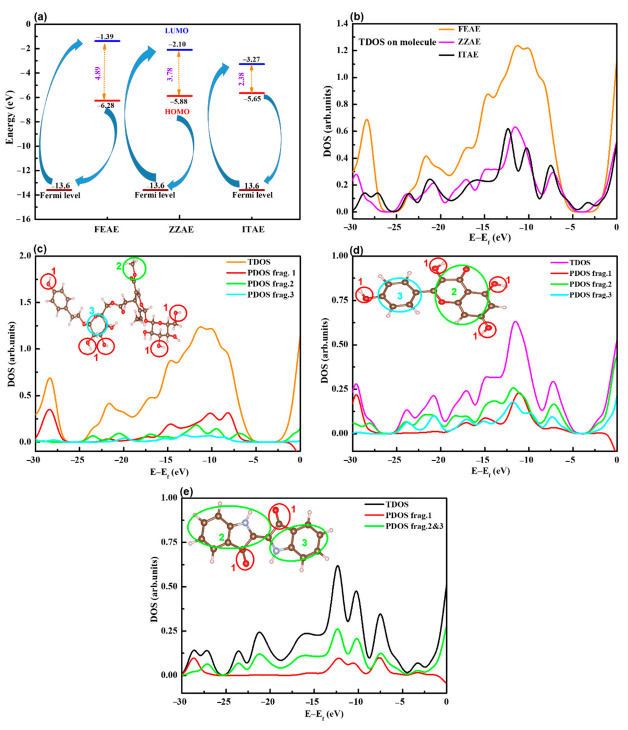
(**a**) HOMO and LUMO energetic positions and energy gap, (**b**–**e**) distribution of total and partial density of states (TDOS and PDOS) for natural products.

The chemical reactivities of the three compounds can be compared based on the energy positions of the HOMO and LUMO, energy gap, ∆E, (Figure 8a), and other quantum descriptors (Table S4). The HOMO and LUMO energies were mainly related to the electron-donating and electron-accepting abilities of the inhibitor molecules. Inhibitor molecules with higher E_HOMO_ and lower E_LUMO_ exhibit potential reactivities for donor–acceptor interactions with metal surfaces. Calculations revealed that the electron donor–acceptor capabilities of the inhibitors followed the order ITAE > ZZAE > FEAE. The same order was obtained while describing the molecular reactivity based on the energy gap. Although the HOMO and LUMO were distributed on a small part of the FEAE inhibitor, its ability to donate electrons did not vary as much as other inhibitors (Figure 7).

A comparison of the QC properties reported in Table S4 shows that the results cannot accurately predict the electron-transfer behavior of the molecules. Overall, this implies that the ITAE and ZZAE inhibitors have higher reactivities than the FEAE inhibitor, considering the smallest ΔE and largest electronegativity (χ) values. However, considering the trend of ΔN (fraction of electrons transferred), the FEAE inhibitor could transfer more electrons to the steel surface and hence presented stronger reactivity. This is not surprising, considering the diverging opinions in the literature regarding the correlation between some QC descriptors of free compounds and their inhibition performance. As shown in Figure 8a, the HOMO energies of the inhibitor molecules are significantly higher than the Fermi level of iron (−13.6 eV), confirming their adsorption ability on the steel surface. However, the possibility of evaluating the inhibitory potential of the inhibitor molecules solely based on the QC properties of isolated molecules must be thoroughly investigated.

The DOS and ELF tools can be used to analyze the nature and properties of the electron structures. The DOS was simulated based on the distribution of the molecular orbital energy levels. Figure 8b shows the total DOS for the three inhibitor molecules, and the results indicate that the relative heights of the curves at different energy regions are in the order FEAE > ZZAE > ITAE, implying that most molecular orbitals in the FEAE system have higher energy than those in other systems (ZZAE and ITAE). In addition, the region around –13 eV (close to the Fermi level of Fe) has an evidently larger state density than other regions, confirming charge transfer between the organic inhibitors and the Fe surface. The contribution of different functional groups can be understood from the partial density of states (PDOS) of the hydroxyl, indoline, phenyl, and formaldehyde moieties in the organic inhibitors (Figure 8c–e). Hydroxyl groups contribute the most to the TDOS, suggesting that O–H and methoxy groups mainly contribute to the bonding character between the molecules and iron orbitals. In the case of the ITAE inhibitor, the two fragments of indoline have comparable contributions. Based on the TDOS and PDOS, we believe that the hydroxyl and methoxy groups primarily act as the active centers, and ITAE has a lower tendency to participate in adsorption due to the absence of sufficient numbers of active centers (particularly O–H groups). These findings were in agreement with the experimental results.

**Figure 9:**
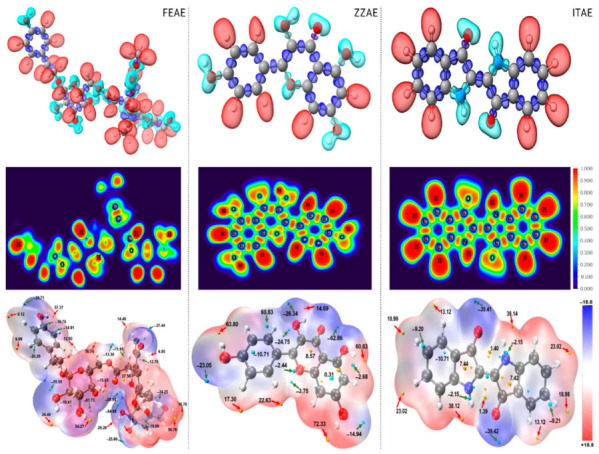
The electron localization function (ELF) isosurfaces (color code: cyan for lone pair domains, red for hydrogen-related domains blue for bonding domains between heavy atoms; isosurface 0.796 a.u.), ELF color-filled maps in the XY plan and electrostatic potential (ESP) mapped vdW surface (color code: orange and blue spheres correspond to ESP maxima and minima on the vdW surface, respectively; isosurface 0.001 a.u.). (ELF isosurfaces: **top**, ELF color-filled maps: **middle row**, ESP: **bottom row**).

ELF was used to reveal the localization and delocalization of electrons in space, which can intuitively reveal the atomic shell structure, chemical bonding, and number of lone-pair electrons. The ELF iso-surfaces and color-filled maps of the investigated inhibitors based on the occupied molecular orbitals are displayed in Figure 9 (top and middle rows). The results suggest that, in addition to the σ bond and π—electron characteristics of the conjugated rings, the O (bonded to H) and N atoms impart the most significant characteristics of lone-pair electrons, thereby rendering a high degree of electron localization. The ELF analysis provided intuitive evidence that FEAE has more mobile lone-pair electrons, reflecting the high ability of the molecule to form coordinate bonds with the MS surface (mainly by O–H). The functional group activity, particularly hydrogen bonding via the hydroxyl groups and lone-pair electrons, helps predict the inhibition performance of the inhibitors based on their interfacial interactions.

Figure 9 (bottom row) also displays the electrostatic potential (ESP) colored van der Waals (vdW) surface along with the surface extrema for each heterocyclic compound to explore the intermolecular interactions and predict the nucleophilic and electrophilic sites in the molecules. The more negative (red region) and more positive (blue region) ESP sites are indicative of electrophilic and nucleophilic reactions, whereas orange and blue spheres correspond to the ESP maxima and minima on the vdW surface, respectively. As shown in the ESP isosurface maps (Figure 9), the negative isosurface regions and minimum points are mainly located at the hydroxyl groups and the lone pair of electrons on the O atoms in the three inhibitor molecules. The results of the ESP distribution around the O–H groups and O atoms suggest that they may act as nucleophilic centers during the adsorption of the inhibitor molecules on the substrate. The O atom in the O-H group had the most negative potential (global surface minimum), with ESPs of −64.88 kcal mol^−1^, −62.86 kcal mol^−1^, and −39.42 kcal mol^−1^ for FEAE, ZZAE, and ITAE, respectively, followed by the O atom in the C=O group. The ESP distribution around the H atom in the O–H group is the global surface maximum, which corresponds to the most positive values. These results indicate that primary bonding with the steel surface is more favorable via hydroxyl groups, which can serve as donors and acceptors for intermolecular hydrogen bonding. The ESP minima around the N atoms in ITAE were not so negative (–2.5 kcal mol^–1^), indicating that ITAE exhibits low reactivity under the present electrolyte conditions. Therefore, the presence of a lone pair of electrons on the O and N atoms is not sufficient to form strong coordinate bonds in the absence of the hydroxyl group. In addition, as there are several hydroxyl groups in a FEAE molecule, the FEAE inhibitor are expected to have higher chemical reactivity and adsorption potential than the ZZAE inhibitor, in addition to higher stability and polarity. This is in agreement with the experimental findings. Therefore, information on electronic properties and intermolecular interactions obtained using ELF and ESP is highly reliable for predicting the adsorption performance of the inhibitors.

#### 3.6.2. Adsorption of Inhibitors on Fe(110) Surface Based on First-Principles DFT Calculations

**Figure 10:**
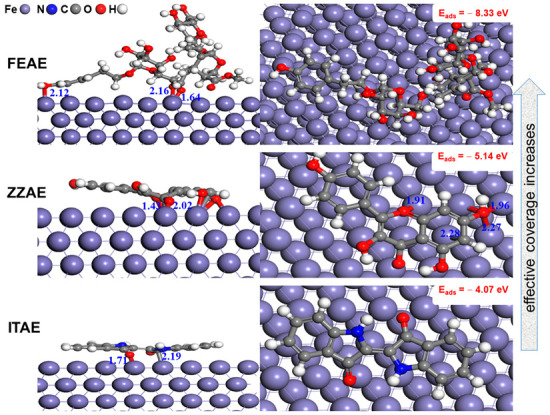
Most stable adsorption configurations modeled for three plant extracts on Fe(110) surface from DFTB calculation. The number on top each plot is the corresponding adsorption energy.

We used first-principles DFT calculations, which can accurately describe the ability to form chemical bonds, to study the possible interactions between the inhibitors and the metal surface. At the molecular level, parallel adsorption configurations have been systematically investigated for their potential to increase the surface coverage of large organic inhibitors [36]. The most stable adsorption configurations of FEAE, ZZAE, and ITAE on the iron surface are shown in Figure 10. Calculations revealed that all three heterocyclic compounds could be adsorbed on the iron surface in a nearly flat arrangement. The FEAE and ZZAE molecules are adsorbed on the Fe surface via hydroxyl groups. In contrast, ITAE molecules bind to the Fe surface via the C and O atoms of the C=O group. The presence of several hydroxyl groups is expected to facilitate strong interactions with the Fe surface, leading to strong coordination behavior. In fact, the presence of hydroxyl groups can influence the adsorption behavior of molecules and intramolecular interactions. The π-current of the phenyl ring in FEAE directly interacts with the Fe surface (Figure 10), suggesting that the O–H group is more active than the C=O group as a donor. Interestingly, the O–Fe bond distance (d_O–Fe_) ranges between 1.64 and 2.12 Å, whereas the Fe–C bond distance in the case of parallel adsorption of the ITAE molecule is 2.19 Å. All measured distances are close to the sum of Fe–O (r_O_ + r_Fe_ = 1.98 Å) and Fe-C (r_C_ + r_Fe_ = 2.08 Å) covalent radii [72]. This suggests that chemisorption and covalent interactions occur between the adsorbed inhibitors and the iron surface via electron transitions between the inhibitor and the Fe 3d band state.

Adsorption energy calculations indicated that the adsorption of FEAE on the Fe surface was energetically more favorable (−8.33 eV) than that of ZZAE (−5.14 eV) and ITAE (−4.07 eV). ITAE did not form covalent bonds with the Fe surface via N atoms, showing less charge transfer between the N sites and the surface. Therefore, the adsorption of the inhibitor molecules is consistent with the high electron localization in the bonding regions, as discussed above, by the ESP-mapped molecular vdW surfaces of the molecules. Likewise, the formation of O–Fe and intermolecular hydrogen bonds are the main driving factors, besides the π-current of the aromatic rings, for the robust interaction between the FEAE and ZZAE inhibitors and the Fe surface. From these results and the structure of the inhibitor molecules, we concluded that a high degree of charge transfer and electron localization enhances the adsorption performance of large inhibitors owing to the sufficient number of lone-pair regions favorable to interactions with iron d-orbitals.

#### 3.6.3. Molecular Dynamics Simulations

**Figure 11:**
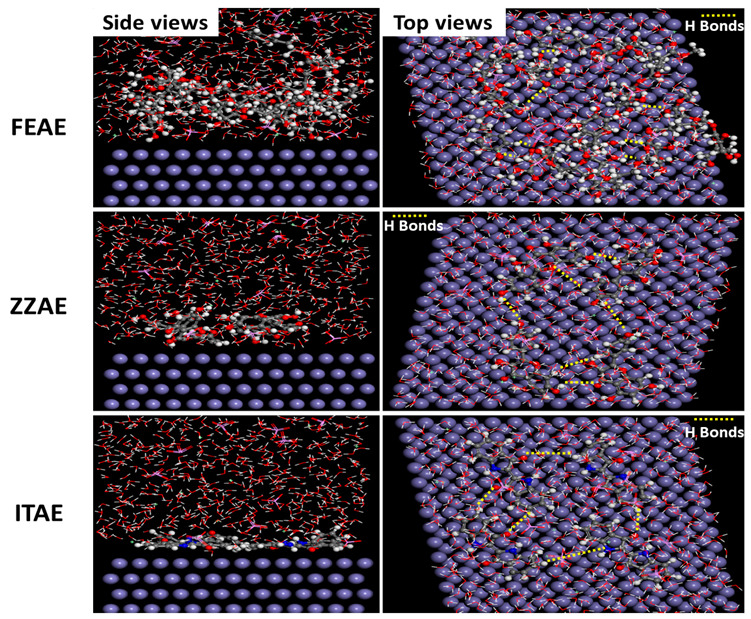
Adsorption process of the plant extracts on Fe(110) in the presence of corrosive species using MD simulations (high symmetry configurations for 4 inhibitor compounds).

The dynamic behavior of the inhibitor molecules on the iron surface in the presence of all corrosive species (H_2_O, H_3_O^+^, and PO_4_^3−^) was explored. The complexity of the systems, the size of the inhibitors, and the diversity of the influencing factors made first-principles simulations difficult. Therefore, MD studies were performed to determine the role of the adsorbed molecules in the solutions, deepening our theoretical understanding of the possible arrangements of inhibitors on the organic-metal interface. The most stable configurations of the adsorbed inhibitors on pure iron in aqueous systems are shown in Figure 11, which also shows the adsorption behavior of all systems containing four molecules and all active species when they reach equilibrium. The results indicate that after desorption, all inhibitor molecules tend to move towards the surface of Fe, where the active centers, particularly the hydroxyl groups, interact directly with the metal surface. Parallel configurations can be considered the result of intermolecular interactions between the inhibitor molecules (top views), on one hand, and the result of mutual attraction between the adsorbed molecules and the iron surface on the other.

Moreover, the three inhibitor molecules formed hydrogen bonds through the O–H and N–H groups. The number of hydrogen bonds between the aqueous FEAE molecules was higher than those of ZZAE and ITAE, confirming that the dense molecular layer on the iron surface originates from the coordinate bonds between the molecules, which eventually interact with the Fe surface. After the final equilibrium, the interaction energy is also an important factor for evaluating the adsorption behavior of the inhibitor molecules. The interaction energies were calculated for four adsorbed inhibitors. The *E_int_* of FEAE (–948.62 kJ mol^–1^) is more negative than those of ZZAE (–589.64 kJ mol^–1^) and ITAE (–514.67 kJ mol^–1^), indicating that FEAE has the strongest adsorption ability and, consequently, covers the largest surface area of Fe. The difference in the interaction energies of the studied systems can also be explained based on the intense interaction between the active sites, especially the hydroxyl groups, and the surrounding medium. The intermolecular interactions and orientation of the inhibitor molecules facilitate charge transfer between the active regions and the Fe surface, which further enhances the corrosion inhibition effect. Therefore, the presence of more active sites in large molecular chains increases the adsorption stability of the inhibitors by forming a dense molecular layer over the metal surface. These results are consistent with those obtained from the first-principles DFT calculations and the experiments.

### 3.7. Comparison with Other Plants Used as Inhibitors for Steels in H_3_PO_4_ Acid

Analyzing various results in the field of corrosion inhibition studies, there is a growing body of literature that recognizes the importance of using plant extracts as corrosion inhibitors for metals and their alloys in different aggressive environments. However, a search of the literature revealed a few studies which report the corrosion inhibition behavior of plant extracts on steel surface in H_3_PO_4_ solutions. In order to give a fair comparison between the studied plant extracts in the present work and those reported in the literature as corrosion inhibitors for steels in H_3_PO_4_ solutions, Table 8 is constructed. Inspection of Table 8 revealed that the studied plant extracts show relatively high performance as compared with that reported in the literature. Thus, the electrochemical performance of the developed organic layer using FEAE, ZZAE, and ITAE extracts is more desirable than those controlled by other organic compounds, as shown in Table 8. Accordingly, these plant extracts represent a good addition to the literature as green corrosion inhibitors of steel corrosion in phosphoric acid solutions due to their high corrosion protection under different conditions.

## 4. Conclusions

Corrosion inhibitors should be chosen not only based on their inhibition efficiency, but also their environmental impact. Despite their high efficiency, many synthetic compounds are not suitable for corrosion inhibition because of the environmental hazards they pose. In this work experimental, DFT, DFTB, and MD studies were conducted on the heterocyclic compounds derived from FEAE, ZZAE, and ITAE to evaluate their action as corrosion inhibitors for mild steel in 2 M H_3_PO_4_, and the following conclusions can be drawn:

- Regardless of the technique used, the inhibition efficiency for mild steel corrosion in 2 M H_3_PO_4_ increased with the inhibitor concentration.
- At 2.5 g L^−1^, the inhibition ability of the plant extracts followed the order: FEAE (95.1 %) > ZZAE (91.7 %) > ITAE (89.4 %), which was confirmed by surface analyses based on SEM and electrochemical measurements.
- The plant extracts act as mixed-type inhibitors by simply blocking the anodic and cathodic active sites without changing the corrosion mechanism.
- The *C_dl_* value decreases significantly with the addition of the extracts, indicating that the thickness of the film adsorbed on the steel surface depends on the concentration of the extracts.
- The SEM and FT-IR techniques confirmed the formation of the adsorbed film. The adsorbed layers exhibited effective anti-corrosion behavior.
- The adsorption of the extracts obeyed the Langmuir adsorption isotherm, and the free energy of adsorption indicated a mixed type of adsorption for the inhibitor species on the metal surface.
- The adsorption of the extracts increased considerably in the parallel mode with the increase in the number of lone-pair electrons (reactive sites) in the molecular structures.
- Direct chemisorptive interactions through the π-current of the aromatic rings and functional groups played a leading role in the stability of the parallel arrangement of the studied inhibitors.

## Figures and Tables

**Table 1:** Chemical structure of the major constituents found in the studied plant extracts [21,22,23,27].

Inhibitor	Chemical Structure	Content (%)
*Fraxinus excelsior* L.	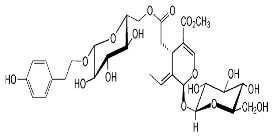	11.42
	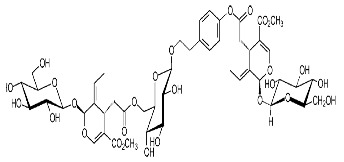	6.15
*Zingiber zerumbet* L.	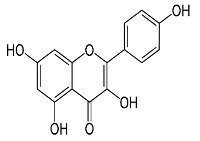	33.60
	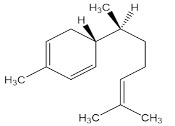	17.40
*Isatis tinctoria* L.	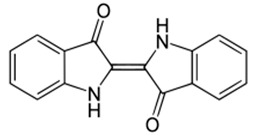	40.00
	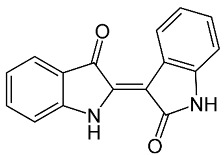	20.89

**Table 2:** Corrosion rates and inhibition efficiencies of mild steel corrosion in 2 M H_3_PO_4_ solution with and without different concentrations of the studied plant extracts.

C_inh_ (g L^−1^)	CR_WL_ × 10^5^ (g cm^−2^ min^−1^)			IE_WL_%		
	FEAE	ZZAE	ITAE	FEAE	ZZAE	ITAE
0.0	5.848 ± 0.014			-	-	-
0.005	3.017 ± 0.013	3.881 ± 0.023	5.219 ± 0.003	48.4	33.6	10.8
0.01	2.387 ± 0.009	3.232 ± 0.034	5.041 ± 0.007	59.2	44.7	13.8
0.025	0.931 ± 0.017	2.478 ± 0.005	3.573 ± 0.012	84.1	57.6	38.9
0.05	0.662 ± 0.005	2.289 ± 0.002	3.293 ± 0.023	88.7	60.9	43.7
0.1	0.577 ± 0.003	2.035 ± 0.012	2.543 ± 0.002	90.1	65.2	56.5
0.25	0.516 ± 0.012	1.924 ± 0.006	2.022 ± 0.005	91.2	67.1	65.4
0.5	0.427 ± 0.022	1.145 ± 0.017	1.396 ± 0.013	92.7	80.4	76.1
1.0	0.342 ± 0.006	0.802 ± 0.008	0.836 ± 0.003	94.2	86.3	85.7
1.5	0.312 ± 0.005	0.631 ± 0.021	0.713 ± 0.009	94.7	89.2	87.8
2.0	0.306 ± 0.016	0.614 ± 0.013	0.678 ± 0.011	94.8	89.5	88.4
2.5	0.286 ± 0.013	0.486 ± 0.015	0.622 ± 0.021	95.1	91.7	89.4
3.0	0.281 ± 0.003	0.420 ± 0.004	0.488 ± 0.004	95.2	92.8	91.7

**Table 3:** Impedance parameters obtained from mild steel corrosion in 2 M H_3_PO_4_ with and without different concentrations of the studied plant extracts at 30 °C.

C_inh_ (g L^−1^)	R_s_ (ohm cm^2^)	R_p_ (ohm cm^2^)	C_dl_ (µF cm^−2^)	CPE “n”	Goodness of Fit (χ^2^) × 10^−3^	IE_R_%
FEAE						
0.0	2.50 ± 0.05	6.94 ± 0.5	365.01	0.99 ± 0.005	1.22	-
0.025	2.81 ± 0.07	18.17 ± 0.8	165.22	0.80 ± 0.006	1.29	61.8
0.05	2.85 ± 0.12	54.13 ± 2.2	88.85	0.81 ± 0.003	3.81	87.2
0.1	2.62 ± 0.06	84.04 ± 2.5	65.30	0.84 ± 0.007	2.44	91.8
0.5	2.50 ± 0.11	120.53 ± 2.4	45.64	0.85 ± 0.006	1.31	94.3
1.0	2.86 ± 0.08	136.43 ± 1.7	33.22	0.88 ± 0.002	2.76	94.9
2.0	2.49 ± 0.07	155.08 ± 2.6	24.12	0.88 ± 0.008	2.14	95.5
2.5	2.38 ± 0.11	181.58 ± 1.9	19.61	0.88 ± 0.004	1.54	96.2
ZZAE						
0.0	2.50 ± 0.05	6.94 ± 0.5	365.01	0.99 ± 0.005	1.22	-
0.025	2.37 ± 0.12	10.05 ± 0.8	280.90	1.00 ± 0.004	1.14	30.9
0.05	3.56 ± 0.08	12.77 ± 0.6	215.23	0.99 ± 0.005	3.21	45.7
0.1	3.54 ± 0.06	16.73 ± 1.1	174.37	0.89 ± 0.002	2.53	58.5
0.5	2.88 ± 0.11	62.52 ± 2.2	94.50	0.83 ± 0.001	1.65	88.9
1.0	2.74 ± 0.13	88.80 ± 2.5	69.43	0.87 ± 0.006	2.39	92.2
2.0	2.69 ± 0.09	138.44 ± 1.8	41.69	0.88 ± 0.008	3.22	94.9
2.5	2.51 ± 0.13	180.93 ± 2.7	26.41	0.88 ± 0.003	2.37	96.2
ITAE						
0.0	2.50 ± 0.05	6.94 ± 0.5	365.01	0.99 ± 0.005	1.22	-
0.025	2.21 ± 0.07	9.85 ± 0.7	291.28	0.99 ± 0.003	3.35	29.6
0.05	3.07 ± 0.12	12.1 ± 0.4	226.37	0.87 ± 0.006	0.81	42.7
0.1	2.69 ± 0.11	15.03 ± 0.9	188.52	0.81 ± 0.002	0.67	53.9
0.5	2.92 ± 0.09	38.11 ± 1.1	122.37	0.82 ± 0.004	1.55	81.8
1.0	2.76 ± 0.06	58.05 ± 1.3	98.63	0.85 ± 0.001	2.08	88.1
2.0	2.84 ± 0.14	68.14 ± 1.1	74.25	0.86 ± 0.002	2.55	89.8
2.5	3.26 ± 0.07	79.04 ± 1.6	52.79	0.86 ± 0.004	2.56	91.2

**Table 4:** Potentiodynamic polarization parameters for mild steel corrosion in 2 M H_3_PO_4_ solutions with and without different concentrations of the studied plant extracts at 30 °C.

C_inh_ (g L^−1^)	−E_corr_ (mV)	i_corr_ (mA cm^−2^)	$\beta_{a}$ $\left(\mathrm{mV}\;{\mathrm{dec}}^{-1}\right)$	$-\beta_{c}$ $\left(\mathrm{mV}\;{\mathrm{dec}}^{-1}\right)$	Rp (ohm cm^2^)	IE_i_%
FEAE						
0.0	473 ± 0.5	3.43 ± 0.05	71 ± 3.5	113 ± 2.4	5.54	-
0.025	453 ± 1.2	1.90 ± 0.03	53 ± 2.3	127 ± 3.5	8.53	44.6
0.05	446 ± 0.7	1.28 ± 0.06	48 ± 2.6	125 ± 3.7	11.72	62.7
0.1	445 ± 1.5	0.81 ± 0.02	50 ± 1.7	126 ± 2.8	19.24	76.2
0.5	438 ± 0.8	0.51 ± 0.03	48 ± 1.2	126 ± 1.3	29.42	85.1
1.0	445 ± 0.3	0.39 ± 0.01	49 ± 2.2	130 ± 2.5	39.79	88.5
2.0	442 ± 1.1	0.32 ± 0.02	49 ± 3.1	131 ± 1.7	48.66	90.6
2.5	431 ± 0.6	0.23 ± 0.01	44 ± 1.4	129 ± 2.2	61.44	93.2
ZZAE						
0.0	473 ± 0.5	3.43 ± 0.05	71 ± 3.5	113 ± 2.4	5.54	-
0.025	467 ± 0.3	2.66 ± 0.02	59 ± 1.6	105 ± 1.3	6.14	22.3
0.05	461 ± 1.2	2.17 ± 0.01	61 ± 1.4	113 ± 2.1	7.91	36.7
0.1	462 ± 1.5	1.69 ± 0.03	57 ± 0.8	103 ± 2.2	9.40	50.8
0.5	449 ± 0.4	0.73 ± 0.01	54 ± 2.1	122 ± 3.5	22.27	78.7
1.0	450 ± 0.2	0.56 ± 0.04	53 ± 1.5	123 ± 1.4	28.63	83.7
2.0	447 ± 1.1	0.39 ± 0.02	48 ± 1.7	122 ± 1.8	38.41	88.6
2.5	446 ± 0.4	0.32 ± 0.01	54 ± 1.1	124 ± 2.6	50.91	90.7
ITAE						
0.0	473 ± 0.5	3.43 ± 0.05	71 ± 3.5	113 ± 2.4	5.54	-
0.025	462 ± 1.1	2.76 ± 0.01	55 ± 0.7	124 ± 2.4	6.01	19.5
0.05	461 ± 0.6	2.34 ± 0.03	60 ± 1.1	122 ± 2.5	7.50	31.8
0.1	459 ± 0.4	1.81 ± 0.02	53 ± 2.1	126 ± 3.3	8.96	47.1
0.5	451 ± 1.3	1.02 ± 0.04	55 ± 1.2	125 ± 2.6	16.27	70.2
1.0	451 ± 0.7	0.70 ± 0.01	52 ± 0.6	128 ± 1.2	22.81	79.6
2.0	448 ± 1.1	0.45 ± 0.03	53 ± 1.3	125 ± 1.1	35.72	86.9
2.5	453 ± 0.3	0.38 ± 0.02	53 ± 1.5	121 ± 1.4	42.35	88.9

**Table 5:** Prominent peaks of FT-IR spectra.

Inhibitor	Frequency (cm^−1^)		Band Assignment
	Plant Extract-Powder	Film Adsorbed on Mild Steel	
FEAE	3395.74	3437.03	O-H stretching
	2926.79	2924.48	C-H stretching
	2855.92	2852.60	
	1662.83	1636.81	C=O (ester), C=C stretching
	1120.22	1113.20	C-O stretching
	1078.14	1059.60	
ZZAE	3421.43	3435.93	O-H stretching
	2925.48	2927.05	C-H stretching
	2854.16	2855.53	
	1639.10	1634.43	C=O (ketone), C=C stretching
	1078.57	1059.73	C-O stretching
ITAE	3401.95	3444.71	N-H stretching
	1603.18	1633.75	C=O (amide), C=C stretching

**Table 6:** Langmuir’s adsorption parameters for the studied plant extracts from 2 M H_3_PO_4_ on mild steel.

Technique		Langmuir Parameters			
	Inhibitor	Slope	*K_ads_* (Lg^−1^)	−Δ*G_ads_* (kJ mol^−1^)	*r* ^2^
FEAE					
WL		1.05	136.42	29.77	0.999
EIS		1.04	98.68	28.96	0.999
PDP		1.07	27.07	25.70	0.999
ZZAE					
WL		1.09	21.90	25.17	0.999
EIS		1.02	16.53	24.46	0.999
PDP		1.07	10.69	23.36	0.999
ITAE					
WL		1.10	15.00	24.22	0.999
EIS		1.07	14.69	24.16	0.999
PDP		1.09	8.05	22.65	0.999

**Table 7:** Corrosion activation parameters for mild steel in 2 M H_3_PO_4_ with and without 2.5 g L^−1^ of the studied plant extracts.

Inhibitor	$E_{a}$ $\left(\mathrm{kJ}\;{\mathrm{mol}}^{-1}\right)$	A (g cm^−2^ min^−1^)	$\Delta H^{*}$ $\left(\mathrm{kJ}\;{\mathrm{mol}}^{-1}\right)$	$\Delta S^{*}$ $\left(J\;{\mathrm{mol}}^{-1}\;K^{-1}\right)$	$E_{a}-\Delta H^{*}$ $\left(\mathrm{kJ}\;{\mathrm{mol}}^{-1}\right)$
Free acid	42.65	103 × 1.35	40.01	−193.88	2.64
FEAE	52.56	103 × 3.35	49.92	−186.29	2.64
ZZAE	52.34	103 × 4.90	49.70	−183.15	2.64
ITAE	57.37	104 × 4.77	54.73	−164.22	2.64

**Table 8:** Quantitative comparison of the inhibition performance of plant extracts under study with the literature data based on plant extracts studied previously as inhibitors in phosphoric acid.

Natural Plant	Metal/Medium	Performance of Optimum Inhibitor Concentration (%)	Reference
Rosemary oil	Steel/2 M H_3_PO_4_	73.0	[15]
Artemisia oil	Steel/2 M H_3_PO_4_	79.4	[16]
Apricot juice	Mild steel/1 M H_3_PO_4_	75.0	[17]
Red cabbage dye	Mild steel/1 N H_3_PO_4_	76.5	[73]
Black tea	High carbon steel/1 M H_3_PO_4_	93.7	[18]
Psidium guajava (guava) leaf	Mild steel/1 M H_3_PO_4_	89.0	[19]
Red onion seeds and peels	Steel/0.75 M H_3_PO_4_	90.0 (s) 74.7 (p)	[74]
Guar gum	Carbon steel/2 M H_3_PO_4_	95.9	[20]
pomegranate peel	Mild steel/2 M H_3_PO_4_	91.6	[75]
Artemisia herba-alba oil	Stainless steel/1 M H_3_PO_4_	88.0	[76]
Pomelo peel	Mild steel/1 M H_3_PO_4_	95.0	[77]
*Fraxinus excelsior* L. seeds	Mild steel/2 M H_3_PO_4_	96.2	Present work
*Zingiber zerumbet* L. roots		96.2	
*Isatis tinctoria* L. leaves		91.2	

## Data Availability

The data related to this work can be obtained from the corresponding authors upon reasonable request.

## Supplementary Materials

The following supporting information can be downloaded at: https://www.mdpi.com/article/10.3390/ma15238688/s1.
