# Supplementary material for: Development of Natural Plant Extracts as Sustainable Inhibitors for Efficient Protection of Mild Steel: Experimental and First-Principles Multi-Level Computational Methods
Source: Materials (Basel). 2022 Dec 6;15(23):8688. doi: 10.3390/ma15238688 (PMC9736516; doi:10.3390/ma15238688)
Supplement: Supplementary file 1 [file materials-15-08688-s001.zip › materials-2018653-supplementary.pdf]

Supporting information to

Development of Natural Plant Extracts as Sustainable Inhibitors for Efficient Protection of Mild Steel: Experimental and First-Principles Multi-Level Computational Methods

Aisha H. Al-Moubaraki [1,†], Abdelkarim Chaouiki [2,*,†], Jamilah M. Alahmari [1], Wesam A. Al-hammadi [3], Ehteram A. Noor [1], Azza A. Al-Ghamdi [1] and Young Gun Ko [2,*]

[1] Department of Chemistry, Faculty of Sciences—Alfaisaliah Campus, University of Jeddah, Jeddah 21589, Saudi Arabia
[2] Materials Electrochemistry Laboratory, School of Materials Science and Engineering, Yeungnam University, Gyeongsan 38541, Republic of Korea
[3] Department of Chemistry, Faculty of Science and Humanities, Shaqra University, Dawadmi 11911, Saudi Arabia
* Correspondence: abdelkarim.chaouiki@yu.ac.kr (A.C.); younggun@ynu.ac.kr (Y.G.K.)
† These authors contributed equally to this work.

In this study, three different plants named *Fraxinus excelsior* L., *Zingiber zerumbet*, and *Isatis tinctoria* L. were used as a new green inhibitor for corrosion of mild steel in H_3PO_4 solutions (Figure S1). A brief description of each of these plants is as follows:

- *Fraxinus excelsior* L., Common ash (Family: *Oleaceae*), is cultivated naturally in North Africa, Europe, and Asia, from the Atlantic Ocean shores in the West to the Volga River in the East. According to several reports, the seeds of *F. excelsior* L. have long been used as a food, condiment, and traditional medicine. In Morocco, the ash tree is referred to as “Lissan Ettir”, and its seeds as “L’ssane l’ousfour”. Ash seeds are used as a carminative

and to dissolve bladder stones in Iran. The anti-oxidative, anti-inflammatory, anti-rheumatic, analgesic, and antipyretic properties of this plant have been reported.

- *Zingiber zerumbet* L. is regarded as an important member of the *Zingiberaceae* family. It is a Southeast Asian native that is now widely planted in tropical and subtropical locations all over the world. Many countries such as India, Nepal, Malaysia, Bangladesh, and Sri Lanka are the key markets for it. This plant is known as 'Asian ginger' or 'bitter ginger. It is also known as 'Pinecone ginger' and 'Shampoo ginger'. The entire parts of the plant are useful in traditional medicines for the treatment of a variety of diseases. They are also suitable antispasmodic, antirheumatic, and diuretic agents.
- *Isatis tinctoria* L. (woad, *Brassicaceae*), has a documented history as a medicinal herb and as an indigo dye in temperate climate zones. Woad's native environment is likely the grasslands of southeastern Russia; nonetheless, the plant was quickly spread throughout Europe and eastern Asia. *I. tinctoria* was the most important indigo dye in Europe from the Middle Ages to the 18th century. However, with the availability of less expensive indigo sources, its significance faded. Several Renaissance and Baroque herbals provide detailed descriptions of its medical effects. Woad was used for wounds, ulcers, tumors, haemorrhoids, snake bites, and a variety of inflammatory conditions. Antiviral, antifungal, antibacterial, and cytoinhibitory properties of *I. tinctoria* extracts and selected components have been investigated.

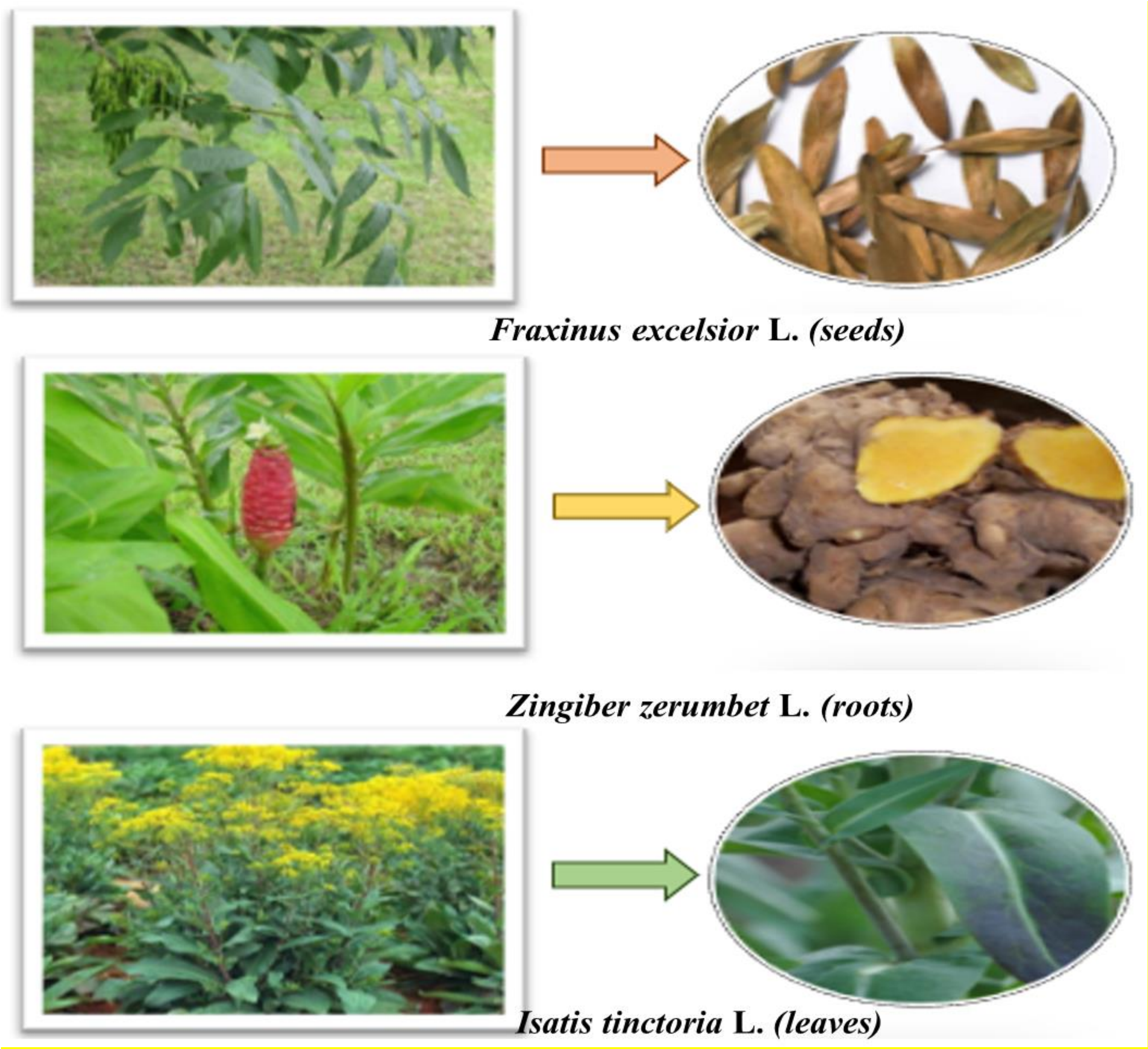

Figure S1. The plants and their parts used in the study.

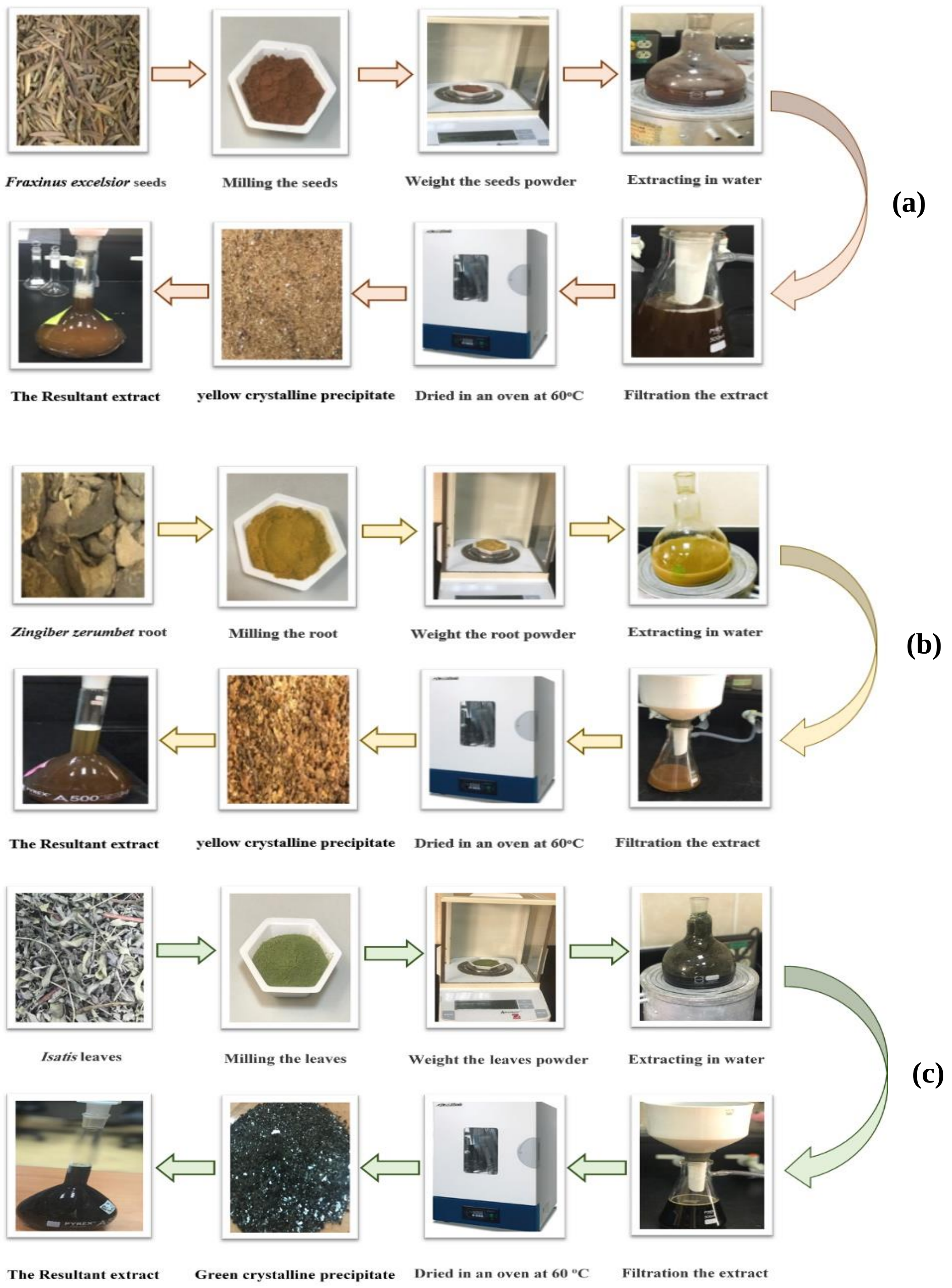

Figure S2. General procedure for the preparation of the extracts (a): *F. excelsior* seeds (FEAE), (b): *Z. zerumbet* roots (ZZAE) and (c) *I. tinctoria* leaves (ITAE).

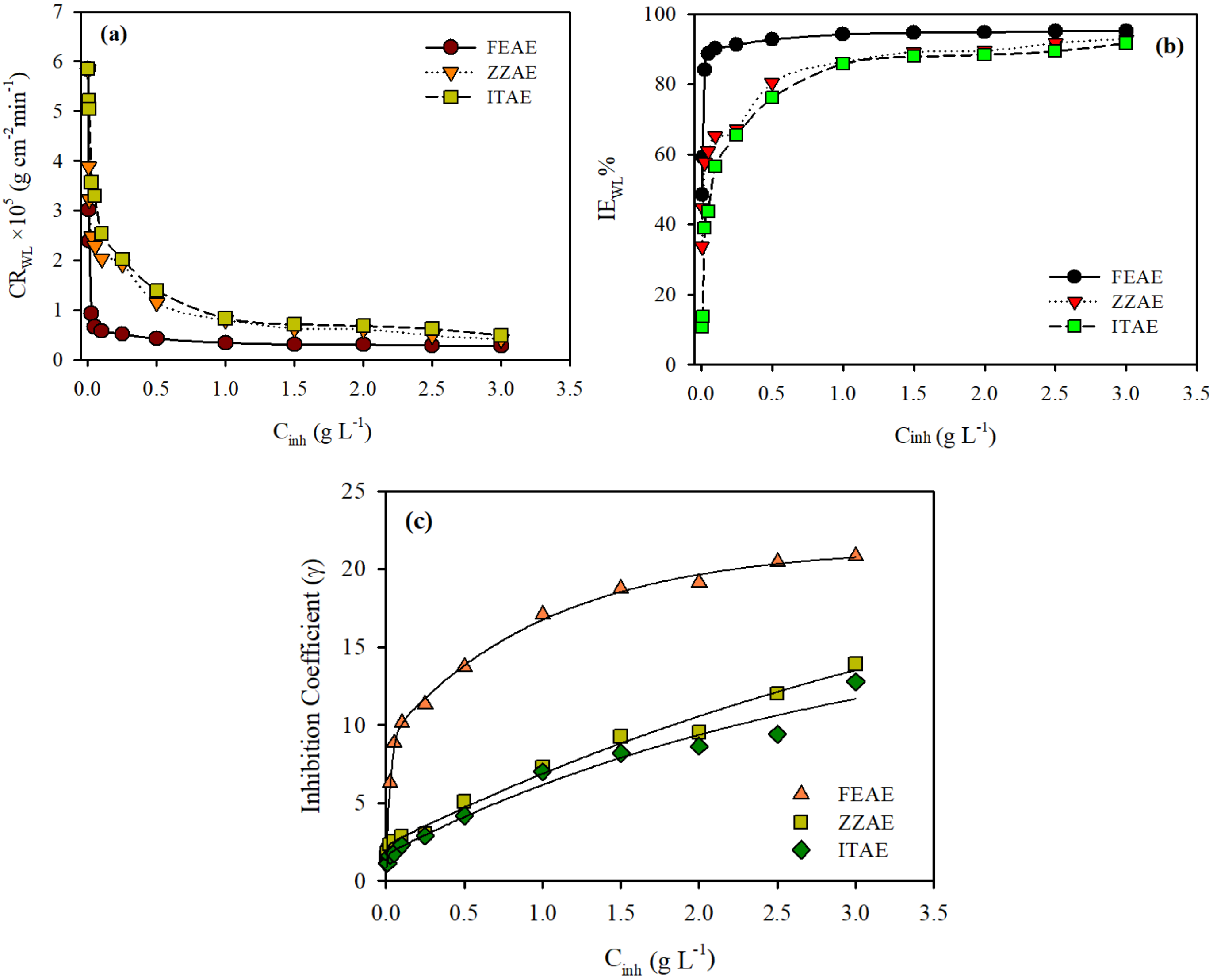

Figure S3. Variation of (a) CR_{WL}, (b) IE_{WL}% and (c) inhibition coefficient (γ) with the concentration of the studied plant extracts for MS in 2 M H_3PO_4 at 30ºC.

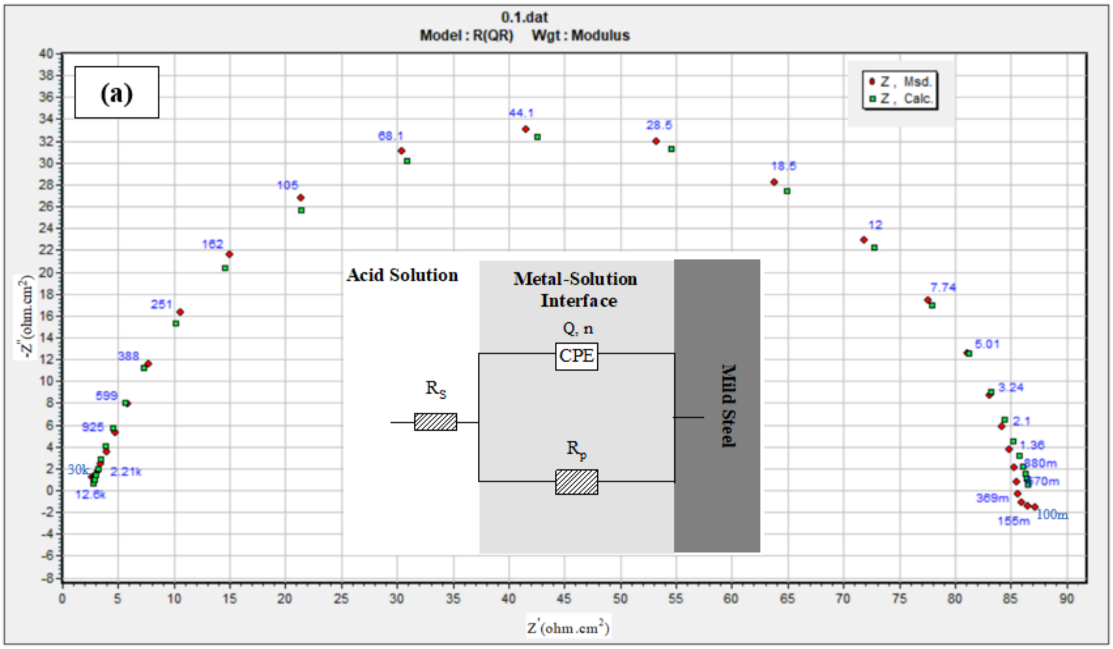

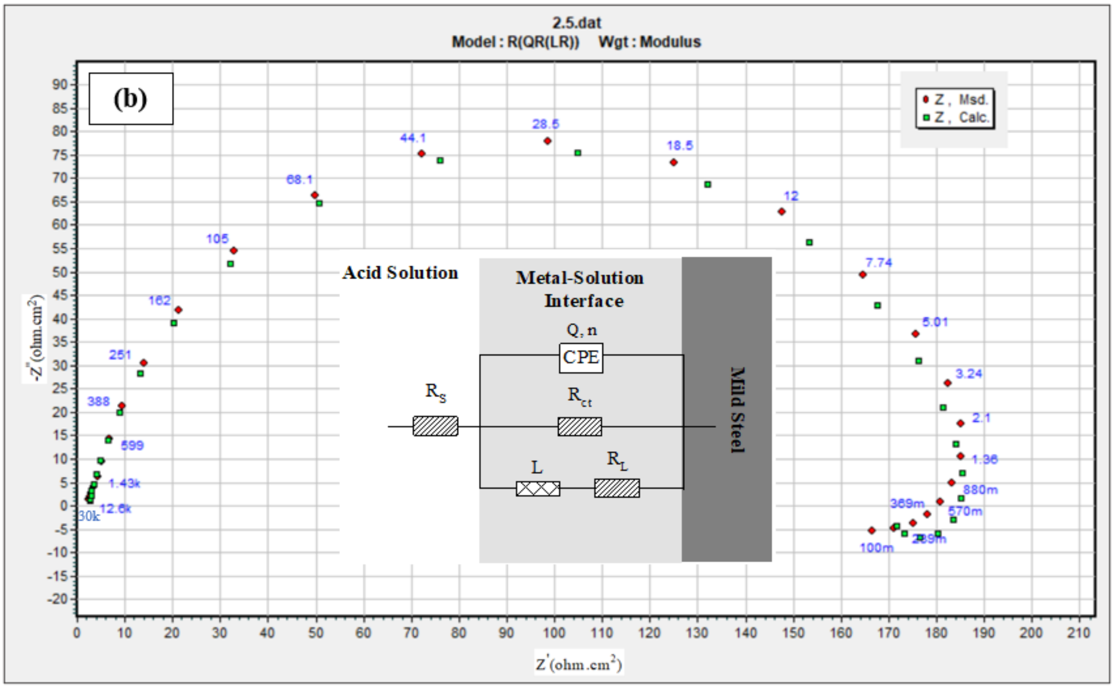

Figure S4. The equivalent circuit models used to fit the impedance data. (a) with and without 0.025–0.1 g L^{-1} of the plant extracts and (b) systems containing 0.5–2.5 g L^{-1} of the plant extracts. (An example for low and high concentration of plant extracts).

Table S1. Some common adsorption isotherms.

Adsorption isotherm model		Eq. No.	plot
	$C_{inh}\,\theta^{-1} = \frac{1}{K_{ads}} + C_{inh}$	**(12-a)**	$C_{inh}\theta^{-1}$ vs C_{inh}
Langmuir	$\frac{\theta}{1-\theta} = K_{ads}\,C_{inh}$	**(12-b)**	$\frac{\theta}{1-\theta}$ vs C_{inh}
	$log(\frac{\theta}{1-\theta}) = log\,K_{ads} + log\,C_{inh}$	**(12-c)**	$\log(\frac{\theta}{1-\theta})$ vs $\log C_{inh}$
Freundlich	$\log\theta = \log K_{ads} + nlog\,C_{inh}$	**(12-d)**	$\log\theta$ vs $\log C_{inh}$
Temkin	$\theta = \frac{1}{f} ln\,K_{ads} + \frac{1}{f} ln\,C_{inh}$	**(12-e)**	θ vs $\ln C_{inh}$
Flory-Huggins	$\log(\frac{\theta}{C_{inh}}) = logK_{ads} + xlog\,(1-\theta)$	**(12-f)**	$\log(\frac{\theta}{C_{inh}})$ vs $\log(1-\theta)$
Frumkin	$ln\left(\frac{\theta}{(1-\theta)C_{inh}}\right) = lnK_{ads} + 2a\theta$	**(12-g)**	$ln\left(\frac{\theta}{(1-\theta)C_{inh}}\right)$ vs θ
Al Awadi Kinetic-thermodynamic	$log(\frac{\theta}{1-\theta}) = log\,K' + y\,log\,C_{inh}$	**(12-h)**	$log\left(\frac{\theta}{1\text{-}\theta}\right)$ vs $\log C_{inh}$

Where C_{inh} is inhibitor concentration; θ is degree of surface coverage; K_{ads} is equilibrium constant of adsorption; *a* is Frumkin constant for lateral interactions between adsorbed inhibitor molecules; f is the Temkin heterogeneity factor (-2*a*); *y* is the number of inhibitor molecules occupying one active site; K' is a constant related to the equilibrium constant of adsorption, K_{ads}, by $K_{ads} = K'^{(1ly)}$; and *x* = (1/*y)* is the number of active sites of the metal surface occupied by one molecule of inhibitor.

Table S2: Correlation coefficients estimation by fitting the results of some isotherm models.

Technique / Inhibitor	Correlation coefficients (r^2)			
	**Langmuir**	**Freundlich**	**Temkin**	**Flory-Huggins**
**FEAE**				
WL	0.999	0.913	0.923	0.997
EIS	0.999	0.563	0.599	0.962
PDP	0.999	0.819	0.884	0.897
**ZZAE**				
WL	0.999	0.992	0.993	0.992
EIS	0.999	0.911	0.956	0.994
PDP	0.999	0.909	0.973	0.998
**ITAE**				
WL	0.999	0.966	0.984	0.996
EIS	0.999	0.926	0.967	0.993
PDP	0.999	0.927	0.990	0.971

Table S3: Corrosion rates and inhibition efficiency of mild steel in 2 M H_3PO_4 with and without 2.5 g L^{-1} of the studies plant extracts at different temperatures.

T (°C)	Corrosion Rate , $CR_{WL}\times 10^5$ ($g\ cm^{-2}\ min^{-1}$)				Inhibition Efficiency (IE_{WL}%)		
	**Free acid**	**FAEA**	**ZZAE**	**ITAE**	**FAEA**	**ZZAE**	**ITAE**
30	5.848±0.014	0.286±0.013	0.486±0.015	0.622±0.021	95.1	91.7	89.4
40	10.469±0.017	0.594±0.006	0.864±0.005	1.280±0.014	94.3	91.7	87.8
50	17.890±0.022	1.055±0.012	1.624±0.009	2.467±0.006	94.1	90.9	86.2
60	26.552±0.025	1.903±0.008	3.163±0.011	4.885±0.007	92.8	88.1	81.6

Table S4: Quantum chemical descriptors of the plant extracts compounds optimized at 6-311++G(d,p).

Molecule	*IE*	*EA*	σ	η	χ	ΔN	ω^+	ω^-
**FEAE**	6.29	1.40	0.40	2.44	3.84	0.10	1.40	5.25
**ZZAE**	5.88	2.10	0.52	1.89	3.99	0.08	2.45	6.44
**ITAE**	5.65	3.27	0.84	1.19	4.46	0.03	6.27	10.73

Values are report in eV.
